# Recent advances in designing synthetic plant regulatory modules

**DOI:** 10.3389/fpls.2025.1567659

**Published:** 2025-04-02

**Authors:** Namitha Nayak, Sandhya Mehrotra, Arti Narendra Karamchandani, Diana Santelia, Rajesh Mehrotra

**Affiliations:** ^1^ Department of Biological Sciences, Birla Institute of Technology and Sciences Pilani, Goa, India; ^2^ Institute of Integrative Biology, ETH Zürich Universitätstrasse, Zürich, Switzerland

**Keywords:** cis-regulatory elements, multigene circuits, synthetic biology, synthetic promoter, synthetic transcription factor

## Abstract

Introducing novel functions in plants through synthetic multigene circuits requires strict transcriptional regulation. Currently, the use of natural regulatory modules in synthetic circuits is hindered by our limited knowledge of complex plant regulatory mechanisms, the paucity of characterized promoters, and the possibility of crosstalk with endogenous circuits. Synthetic regulatory modules can overcome these limitations. This article introduces an integrative *de novo* approach for designing plant synthetic promoters by utilizing the available online tools and databases. The recent achievements in designing and validating synthetic plant promoters, enhancers, transcription factors, and the challenges of establishing synthetic circuits in plants are also discussed.

## 1 Introduction

With the rising population and the adverse effects of climate change, maintaining agricultural productivity will rely on combating environmental stresses through crop engineering. Synthetic biology aims to address these challenges by introducing multigene circuits in plants. Synthetic multigene circuits comprise non-native DNA that can modulate gene expression in response to external cues or intracellular signaling events and have potential applications in cellular control, reprogramming, perturbation, and outcome reporting (Szenk et al., 2020). However, reliable circuit functioning requires precise and predictable gene regulation (Roell and Zurbriggen, 2020). Natural plant *cis*-regulatory modules (CRMs) (including *cis*-regulatory elements, promoters, transcriptional enhancers, silencers, and insulators) are complex in function and architecture. Identifying plant CRMs is also challenging as they can occur tens or hundreds of kilobases away from the target genes (Li et al., 2019; Lu et al., 2019; Ricci et al., 2019). Schmitz et al., 2022 examine the reporter assays developed to characterize natural plant CRMs. Despite these advances, successful characterization requires epigenetic maps from different cell types and growth conditions (Schmitz et al., 2022). Synthetic regulatory elements offer an alternative to help meet the challenge of precision gene control in multigene circuits. This review details the techniques used to design and validate synthetic inducible plant promoters with brief discussions on synthetic transcription factors and enhancers.

## 2 Promoter

**Figure 1:**
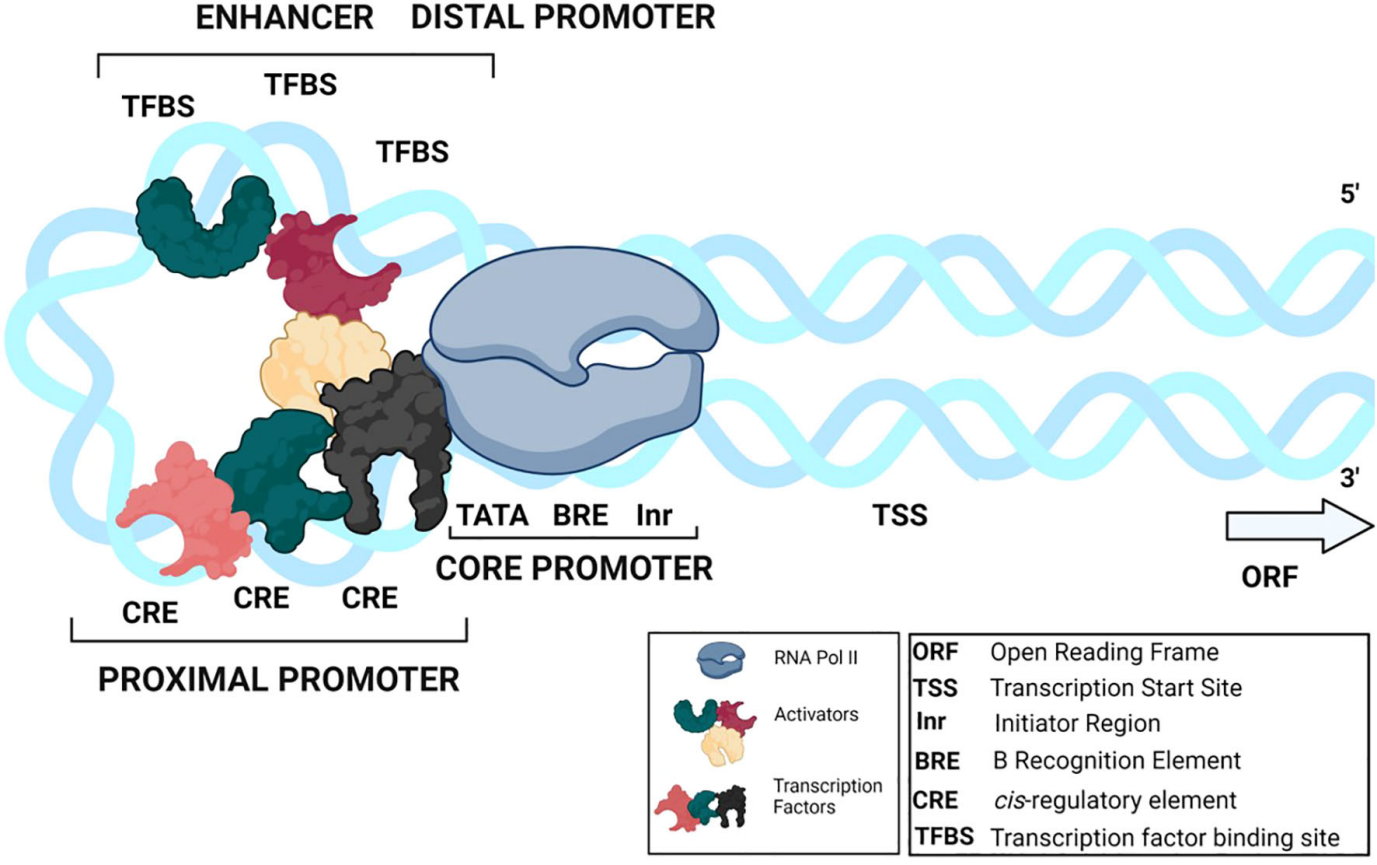
A typical promoter construct A promoter is divided into three regions: the core, proximal, and distal promoter. The core promoter consists of core promoter elements (TATA-box, Inr, BRE). The proximal promoter is comprised of CREs and the distal promoter contains enhancer and silencer elements (TFBSs). General TFs and activators/repressors bind to the CREs and TFBSs, forming a complex along with RNA pol II.

We begin this discussion with an overview of the architecture and function of promoters. A promoter controls the spatial and temporal expression of a gene by binding the RNA polymerase and various transcription factors (TFs) (**Figure 1**) (Vanaja and Yella, 2022). TFs identify short conserved sequences called *cis-*regulatory elements (CREs) in the promoter’s proximal and distal regions (Biłas et al., 2016). CREs and associated TFs are molecular switches of gene regulation that decide the transcriptional activation, transcription efficiency, transcript level per cell, and tissue-specific or condition-specific gene expression (Bhattacharjee et al., 2013). Therefore, screening for known CREs in promoters provides insights into the promoter’s functionality, the potential role of the downstream gene, and the types of TFs involved. However, the gene expression profiles of a genome far outnumber the CREs and TFs present, suggesting that the limited regulatory factors in a cell control the expression of multiple genes through a combinatorial approach, with each promoter employing multiple CREs (Bhattacharjee et al., 2013; Mehrotra et al., 2011).

Based on their activity, promoters are classified as constitutive or inducible. Constitutive promoters steadily express the gene under diverse conditions, while inducible promoters initiate transcription in response to specific cues (Roell and Zurbriggen, 2020). Driving transgene expression with constitutive promoters can result in unintended genetic and phenotypic effects (Bhatnagar-Mathur et al., 2007). Therefore, a stimulus- or tissue-specific expression of transgenes is desirable. Several natural inducible promoters have been characterized and successfully used in heterologous systems without pleiotropic effects (Boni et al., 2018; Misra and Ganesan, 2021; Pandey et al., 2019; Yang et al., 2020). However, multigene circuits require a variety of promoters having a range of strengths and specificities, which at present cannot be satisfied by the limited pool of characterized natural plant promoters.

## 3 Synthetic promoter

The modular nature of promoters and our understanding of CREs under different stresses have enabled the development of synthetic promoters with specific strengths and inducibility.

A synthetic promoter consists of a core promoter with a minimal arrangement of CREs upstream. This concise nature offers many advantages, especially in multigene circuits. Engineered circuits are evolutionarily and genetically unstable (Sleight et al., 2010). Homologous recombination and loss-of-function deletions commonly occurring in repeat sequences of natural promoters are significant causes of network failure (Sleight et al., 2010). Synthetic promoters can be designed to have minimal repeat sequences and high sequence diversity by including functionally equivalent CREs from diverse organisms (Yasmeen et al., 2023). Their low homology to the native genome improves the genetic stability of engineered circuits (Khadanga et al., 2021; Gupta et al., 2021; Peramuna et al., 2018). A multigene circuit’s optimal functioning requires the coordinated expression of multiple genes coupled with precise and predictable transcriptional outputs (Brophy et al., 2022). Computationally designed minimal synthetic promoters and orthogonal TFs with predictable outputs in plants have been developed (Cai et al., 2020).

Synthetic promoters are used in enzymatic pathways to produce biological products. In *Saccharomyces cerevisiae*, synthetic promoters created from the native Translation Elongation Factor 1 (TEF1) promoter were successfully used for efficient glycerol production (Tang et al., 2020). Several synthetic promoters are designed to offer constitutive, inducible, bidirectional, and tissue-specific expression in plants (Jameel et al., 2020; Yang et al., 2021). Cell-state-specific promoters that activate gene expression under specific biological conditions are necessary for many biotechnological applications, including gene therapy (Wu et al., 2019). A suggested method to develop cell-state-specific promoters involves integrating Next-Generation Sequencing (NGS) with Machine Learning (ML) to analyze synthetic promoter libraries and identify high-performance promoters with enhanced cell-state specificity (SPECS) demonstrating distinct spatiotemporal activity (Wu et al., 2019).

## 4 Techniques to generate a synthetic promoter

**Figure 2:**
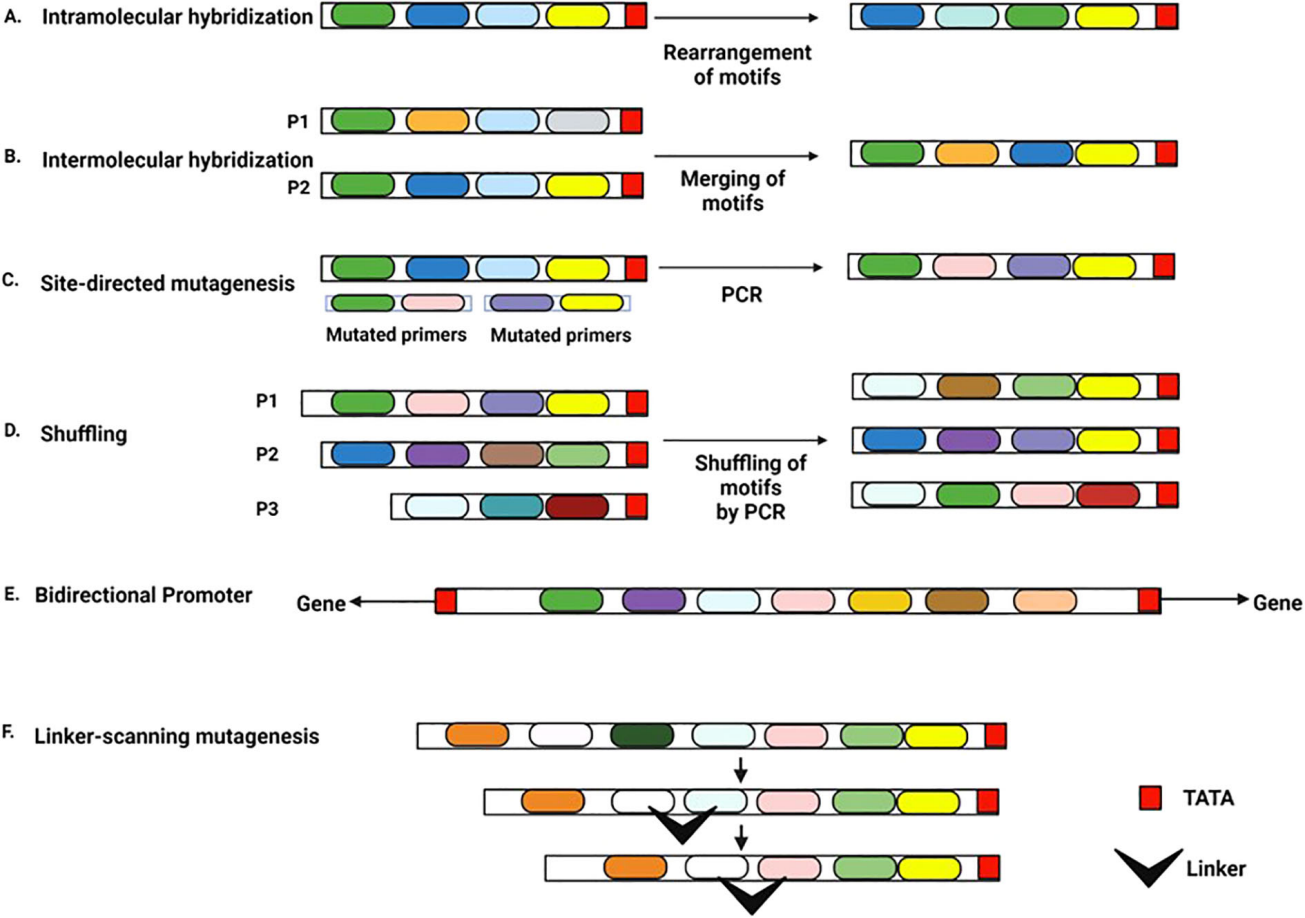
Approaches to generate a synthetic promoter Each colored box represents an individual CRE **(A)** Intramolecular hybridization approach: CREs within a natural promoter are re-arranged to make a new promoter **(B)** Intermolecular hybridization approach: CREs from different natural promoters are combined to form a new promoter, P1 and P2 indicate two different promoters **(C)** PCR-based site-directed mutagenesis approach: primer sequences with rearranged CREs or new CREs can be used to create new promoters in a PCR-based reaction **(D)** Shuffling/ recombination approach: Two or more promoter sequences are cleaved by DNaseI into short fragments (P1, P2, P3) and then reassembled by DNA polymerase into new promoters **(E)** Bidirectional promoter: Two core promoter elements are placed in opposite directions at the ends of a CRE assembly. The promoter regulates two genes situated in opposite orientations **(F)** Linker-scanning mutagenesis: A synthetic linker sequence containing a series of point mutations replaces a region of the same length in the parent promoter.

A synthetic promoter is constructed through a novel arrangement of CREs from different natural promoters or by re-organizing CREs within a promoter (Aysha et al., 2018). For example, artificial Cauliflower Mosaic Virus 35S (CaMV35S) promoters have been synthesized by domain swapping with heterologous promoters or by replacing the native DNA with a synthetic stretch of CREs in a different context (Bhullar et al., 2007). *In vitro* techniques to engineer synthetic promoters include hybridization, site-directed mutagenesis, shuffling, linker scanning mutagenesis, and bi-directionalization (**Figure 2**) (Aysha et al., 2018).

The hybridization approach has two types: intramolecular and intermolecular (Gustin and Burk, 2000). The intramolecular approach rearranges two or more crucial motifs within a promoter (**Figure 2A**). In contrast, the intermolecular approach links key motifs from different promoters to generate a new promoter (Acharya et al., 2014; Gustin and Burk, 2000) (**Figure 2B**). In site-directed mutagenesis, mutations introduced in the natural promoter add or remove specific CREs (Acharya et al., 2014) (**Figure 2C**). Site-directed mutagenesis also helps identify and analyze CREs of natural promoters (Ranjan et al., 2012). Shuffling comprises nucleic acid rearrangements *in vitro*. In a polymerase chain reaction, short segments of two or more promoters are re-joined. The re-assembled promoters contain segments of various promoters, creating a collection of synthetic promoters (Acharya et al., 2014) (**Figure 2D**). Ranjan et al., 2011 generated a library of synthetic promoters from FMV (Figwort Mosaic Virus) full-length and sub-genomic transcript promoters by DNA shuffling. Linker-scanning mutagenesis re-organizes the promoter sequence. The target DNA is replaced with a synthetic linker sequence of similar length containing the desired cluster of point mutations (Gustin and Burk, 2000) (**Figure 2F**). With equal-sized heterologous sequences replacing segments of native DNA, linker-scanning mutagenesis helps analyze entire regulatory regions (Gustin and Burk, 2000). Although mainly applied to examine promoters and enhancers, linker-scanning mutagenesis is also used in synthetic promoter designing. Bi-directional promoters are located between two adjacent genes oriented in opposite directions (Bai et al., 2020) (**Figure 2E**). Such promoters facilitate the simultaneous expression of bidirectional gene pairs situated on opposite DNA strands and lying head-to-head. Synthetic bidirectional green tissue-specific promoters have been designed and validated in transgenic rice (Bai et al., 2020). Khan et al., 2023 have reviewed synthetic plant promoters generated in recent years using the above-mentioned techniques.

Targeted genome editing techniques can modify natural promoters by altering specific CREs *in vivo* and provide fundamental insights into the complex architecture of gene regulatory sequences, upregulate beneficial gene expression, or improve disease resistance (Liu et al., 2021; Oliva et al., 2019; Peng et al., 2017; Rodríguez-Leal et al., 2017). Targeted editing can also bring a gene under the control of an upstream promoter by deleting the intermediate sequence (Bhunia et al., 2022). The basis of targeted genome editing is a sequence-specific nuclease, which is engineered by fusing a non-specific nuclease domain to a customized sequence-specific DNA binding domain (DBD) (Bogdanove et al., 2018; Zetsche et al., 2015). Sequence-specific nucleases include zinc-finger (ZF) nucleases, meganucleases, transcription activator-like effector (TALE) nucleases, and Clustered Regularly Interspaced Short Palindromic Repeats/CRISPR-Associated protein (CRISPR/Cas9) endonuclease (Bogdanove et al., 2018; Zetsche et al., 2015).

## 5 *De novo* approach to construct and validate a synthetic promoter

The *in vitro* and *in vivo* techniques discussed in section 4 generate synthetic promoters by modifying or re-arranging natural promoters. This section discusses a methodology to build inducible synthetic promoters from scratch using the available databases and *in-silico* tools. The designed promoters can be artificially synthesized and validated through transient expression assays.

### 5.1 Screening natural promoters for functional CREs

**Figure 3:**
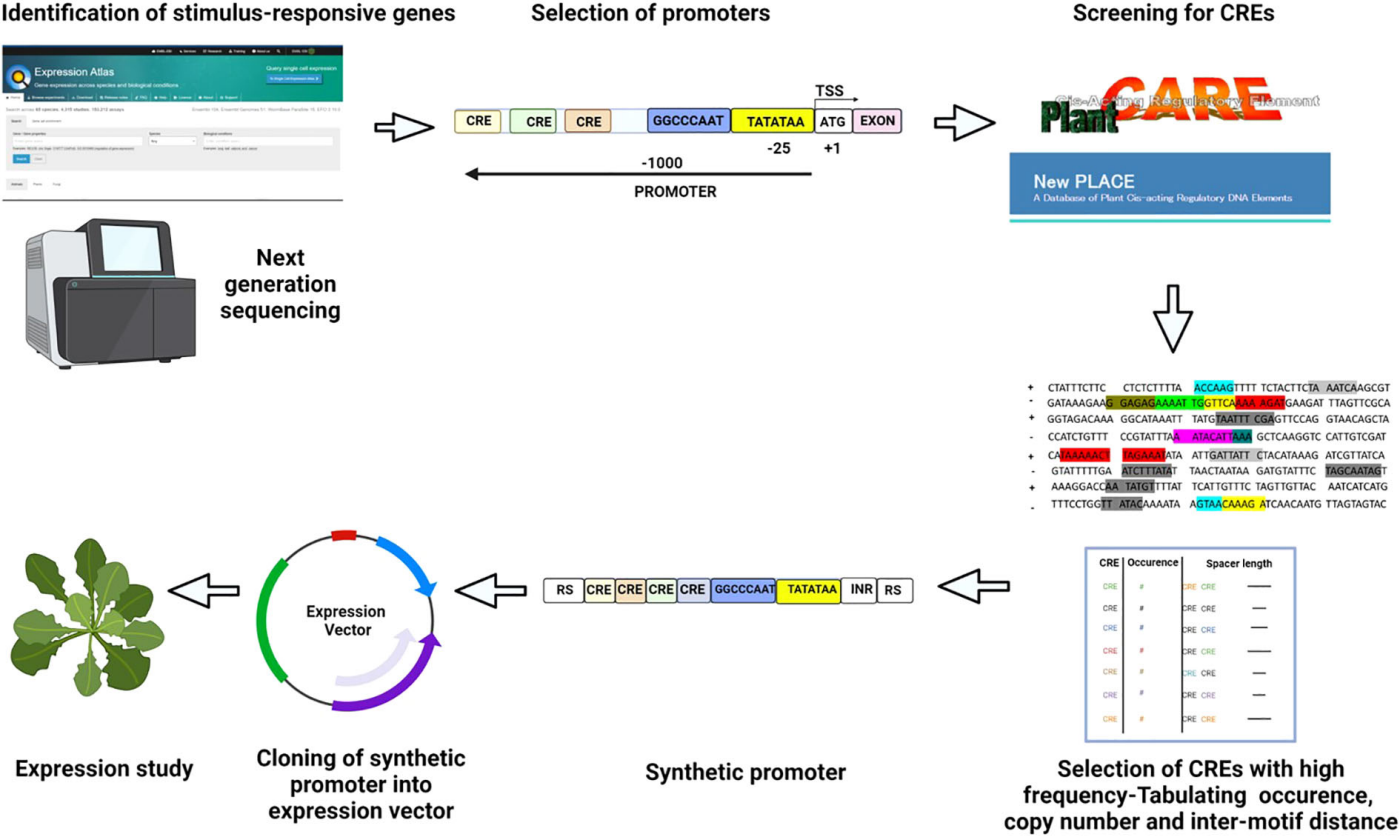
Construction and validation of synthetic promoter Genes upregulated/downregulated in a particular condition are identified using available databases. Promoters for the selected genes are identified using bioinformatic tools, or the 1000 bp upstream region from TSS is marked as the promoter. CREs present in the promoters are screened using PLACE and PlantCARE. The location, copy number, arrangement, and inter-motif distance of selected CREs are decided by analyzing their positions in natural promoters. A synthetic module is designed with core promoter elements (TATA box) and suitable sites for cloning. The synthesized promoter is cloned into an expression vector, and the expression is validated through transient studies. The colored boxes represent individual CREs. RS, Restriction enzyme site; INR, Initiator; TATATAA, TATA box; TSS, Transcription start site.

To design an inducible synthetic promoter, endogenous genes that are overexpressed under a particular stimulus are identified through the microarray expression atlas and NGS databases (**Figure 3**). The promoter sequences of the short-listed genes can be identified using predictive algorithms such as the TSSPlant tool or plant promoter databases like PlantProm (Shahmuradov et al., 2017). Alternatively, a 1-kb region upstream of the gene’s TSS is designated as the promoter (**Figure 3**).

Prior to promoter analysis, it is necessary to identify the probable CREs and TFs acting in a particular stimulus. CRE databases such as New Plant *cis*-acting regulatory DNA elements (PLACE) (Higo et al., 1999), Transcription factor database TRANSFAC (Matys et al., 2003), and Plant *cis*-acting regulatory elements, enhancers, and repressors (Plant CARE) (Lescot et al., 2002) can be employed (**Figure 3**). Promoters of characterized genes can be screened for CREs using *de novo* motif discovery tools. The Hypergeometric Optimization of Motif Enrichment (HOMER) tool identifies over-represented motifs, including minor variants to the consensus CRE (Heinz et al., 2010). The Spaced Motif analysis (SpaMo) tool detects spacer-separated CRE pairs and checks for co-occurring CREs (Whitington et al., 2011), while the Motifs Co-occurrence tool (MCOT) accounts for CRE structure and recognizes overlapping motifs (Levitsky et al., 2019). Conserved CREs that are important for plant development can be identified by phylogenetic footprinting, a method that superimposes sequence conservation data on the regulatory code (Zemlyanskaya et al., 2021). Short, conservative, non-coding plant sequences and genome-wide conservation profiles are available in PLAZA (Van Bel et al., 2018) and Plant Transcriptional Regulatory Map (PlantRegMap) databases (Tian et al., 2020), respectively. Plants have short conserved regulatory regions, which makes it difficult to detect conservation among distantly-related genomes. The Conservation of Motif Variants (CoMoVa) tool offers an alignment-free solution for the detection of small degenerate sequences in known motifs over large evolutionary distances (Lieberman-Lazarovich et al., 2019). ML methods are also promising for identifying CREs from whole-genome annotation data (Eraslan et al., 2019; Wang et al., 2020). ML uses a suite of well-annotated genomic data as training sets to directly infer regulatory signatures. The predictive power of the generated model is then evaluated using independent testing data sets (Eraslan et al., 2019; Zemlyanskaya et al., 2021). ML has been used to predict the regulatory composition and evaluate the transcriptional responses to stress in plants (Mejía-Guerra and Buckler, 2019; Uygun et al., 2019). Since a *cis*-element has a core sequence of 4–10 base pairs, detecting CREs in a 500–5000 base pair-long promoter region is difficult. It is necessary to distinguish between over-represented motifs and background noise. One widely established model to identify functionally relevant motifs is to group genes based on their expression profiles and detect over-represented CREs within the promoters of each group (Sinha et al., 2008). Multiple Expectation maximizations for Motif Elicitation (MEME) and Gibbs sampling are computationally robust tools that identify and evaluate over-represented motifs within promoters (Lambrughi, 2015). **Table 1** summarizes the databases and tools that can be utilized for CRE detection. A broadly effective synthetic promoter can be designed by selecting over-expressing genes from monocots and dicots and listing common CREs. All the identified CREs should be listed, and the copy number and spacer length between motifs should be calculated (**Figure 3**).

**Table 1:** Databases and tools for CRE detection.

Database/Tool	Application
PLACE	Plant CRE database
TRANSFAC	Transcription factor database
Plant CARE	Plant CRE database
HOMER	Identification of over-represented motifs
SpaMo	Identification of co-occurring motifs
MCOT	Identification of overlapping CREs
PLAZA	Platform for plant comparative genomics
PlantRegMap	Database for plant transcription factors, regulatory elements, and interactions
CoMoVa	Detection of conserved motifs across species
MEME	Identification of over-represented motifs
Gibbs sampling	Identification of over-represented motifs

### 5.2 Building a synthetic promoter

#### 5.2.1 Importance of promoter architecture

The type, location, copy number, orientation, and arrangement of CREs form the premise for an effective synthetic promoter. The CRE flanking sequences and the chromatin state also influence the type of TF bound (Mehrotra et al., 2011). Here, we briefly discuss the CREs involved in plant abiotic stress to understand the importance of CRE arrangement in promoter strength and specificity. Abiotic-stress inducible promoters commonly contain the Dehydration-Responsive Element (DRE)/C repeat element (TACCGACAT/TGGCCGAC) and the abscisic acid-responsive element (ABRE) (PyACGTGGC) (Joshi et al., 2016). Other abiotic stress-responsive CREs include the W-box element (TGAC), G-box element (CACGTG), MYC (CAC(GA) TG), LTRE (CCGAC), and MYBR (C/TAACNA/G) (Joshi et al., 2016).

**Figure 4:**
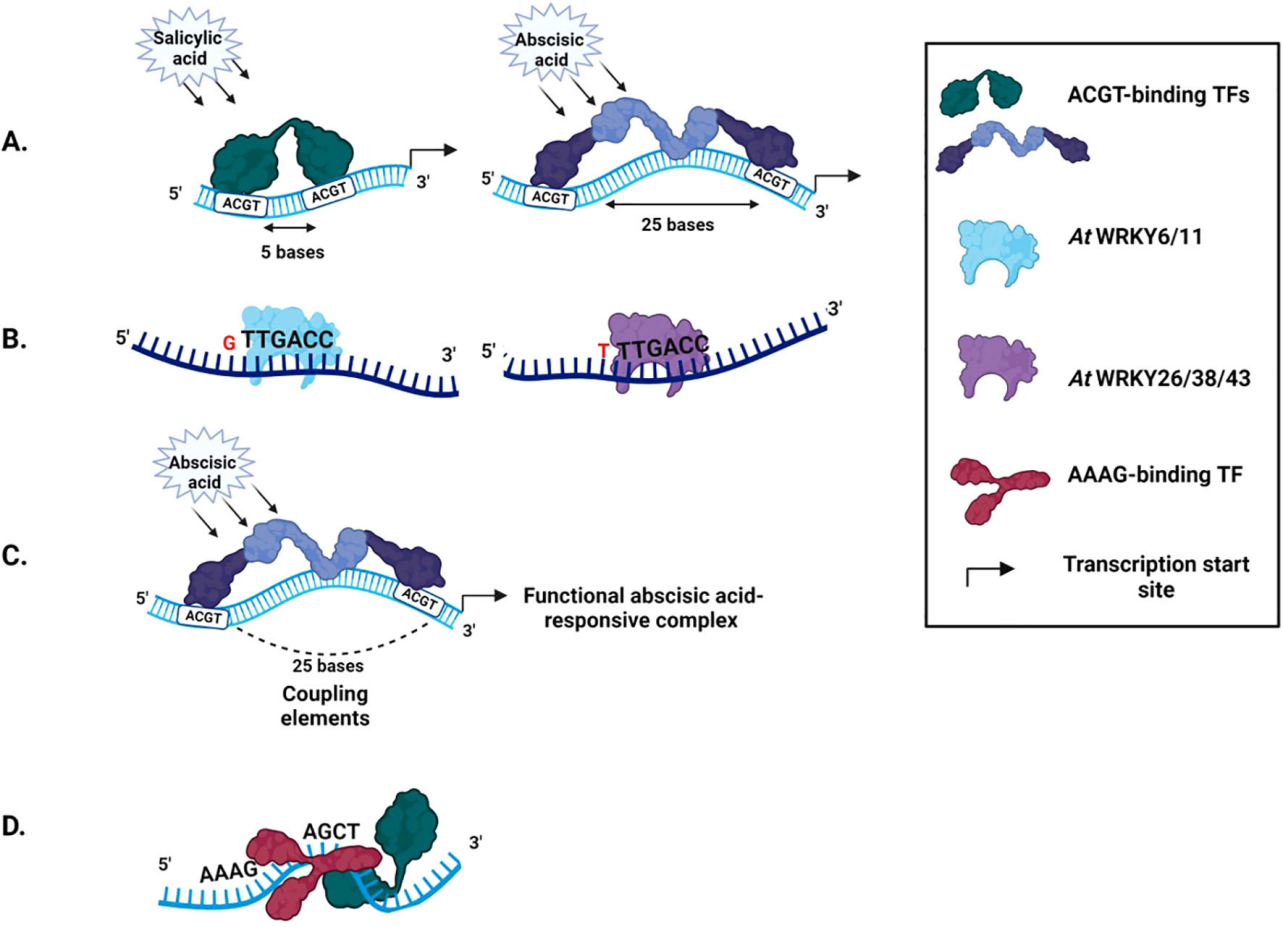
*Cis*-regulatory elements in a promoter **(A)** Spacer length defines TF binding. A distance of 5 bases between two ACGT motifs leads to salicylic acid inducibility, while a distance of 25 leads to abscisic acid inducibility **(B)** Spacer sequences influence the type of transcription factor recruited. *At*WRKY6 binds well to the W-box motif having a G residue at the 5’ end, while *At*WRKY 26 prefers a T, C, or A residue **(C)** Coupling elements. Multiple ABREs couple to form a functional abscisic acid-responsive complex **(D)** Coupling elements can occur in a specific orientation in plants. 5’AAAG(n)ACGT3’ is the preferred orientation over 5’ACGT(n)AAAG3’ in *Arabidopsis*.

Abiotic stress-responsive CREs display a preference for particular spacer lengths. The W-box motif pattern in *Arabidopsis* favors 3-4 nucleotides spacing and shows a lack of preference for consecutive TGAC motifs (Dhatterwal et al., 2021). Analysis of spacer frequency between combinations of the DRE and MYC-like motif with that of the ACGT motif across *Arabidopsis*, rice, wheat, and soybean genomes show a clear preference for particular spacer lengths (Bhadouriya et al., 2021). The predominance of these spacer lengths is likely related to the increased stability provided by helical phasing (Mehrotra et al., 2013). Transcription factors favor specific spacer lengths (Dhatterwal et al., 2021). A spacer distance of 5 nucleotides between two ACGT motifs confers salicylic acid inducibility to a minimal promoter. However, increasing the distance to 25 nucleotides confers abscisic-acid inducibility (Mehrotra and Mehrotra, 2010) (**Figure 4A**).

The type of TF recruited at a CRE can be determined by the nucleotides neighboring the core motif, which translates to terminal base conservation in native spacer sequences (Bhadouriya et al., 2021). The TFs *At*WRKY6 and *At*WRKY11 bind well to W-boxes having a G residue at the 5’ end, while TFs *At*WRKY 26, 38, and 43 prefer a T, C, or A residue (Ciolkowski et al., 2008) (**Figure 4B**).

CRE functioning can depend on its copy number, orientation, and the cell-type. Co-occurring CREs are termed Coupling Elements (CE). A single copy of ABRE cannot induce transcription. Multiple ABRE copies or ABRE coupled with DRE, CE1, and CE3 form a functional abscisic acid-responsive complex (Narusaka et al., 2003; Shen et al., 1996) (**Figure 4C**). CRE positioning within the promoter can impact the assembly of the transcription machinery. Therefore, certain coupling motifs are found in specific orientations. In *Arabidopsis*, AAAG(n)ACGT is the preferred orientation over ACGT(n)AAAG (Khan et al., 2022) (**Figure 4D**). Some CREs act in a tissue-specific manner. The AACA motif acts as a positive regulatory element in the endosperm but a negative regulatory element in other tissues (Bhalothia et al., 2013).

#### 5.2.2 Designing the proximal promoter

A trial-and-error approach is adopted to design synthetic promoters using selected motifs. Since TFs bind to CREs in a particular fashion, random order and spacing between motifs can reduce expression strength (Mehrotra et al., 2017). The copy number and spacer length of CREs in the synthetic promoter are decided based on the preference observed in candidate native promoters (Bhadouriya et al., 2024) (**Figure 3**). As flanking sequences affect TF recruitment, maintaining native spacer sequences is beneficial (Ghoshdastidar and Bansal, 2022). However, native spacers can include unknown CREs that affect the expression profile of the synthetic promoter. A potential solution is to analyze native spacers for conserved flanking nucleotides, which can then be placed in a randomly generated spacer to reduce background expression (Cai et al., 2020). A collection of synthetic promoters can be manually designed by an organized arrangement of selected motifs in the proximal promoter. Alternatively, a computational approach can be employed to design a synthetic promoter library by selecting an appropriate background nucleotide frequency (based on the plant system) and incorporating core CREs with a randomized arrangement of stimulus-determinant CREs in the proximal region (Cai et al., 2020; Jores et al., 2021).

#### 5.2.3 Designing the core promoter

Core promoter elements complete the synthetic promoter. The GC content of core promoters influences the transcription strength depending on the plant system (Eraslan et al., 2019; Wang et al., 2020; Zemlyanskaya et al., 2021). For example, core promoters with high GC content are less effective in a tobacco assay system as their transcriptional machinery is attuned to AT-rich promoters (Jores et al., 2021).

The TATA box, B Recognition Element (BRE), Y patch, and Initiator (Inr) strongly influence promoter strength (Jores et al., 2021). Core promoters having a TATA box, especially within 23 to 59 base pairs upstream of the TSS, were four-fold stronger than TATA-less promoters in *Arabidopsis*, maize, and sorghum (Jores et al., 2021). Introducing operator sequences in the regions flanking the TATA box repressed the *Arabidopsis Translationally Controlled Tumor Protein (TCTP)* promoter effectively. Variations in the TATA box- flanking operator sequence resulted in a range of expression levels (Jores et al., 2021). Khan et al. (2024) engineered a promoter library displaying a range of expression levels by replacing the TATA box flanking sequences in the *TCTP* and *CamV35S* promoters with short guide RNA (sgRNA) binding sites. In maize, an upstream BRE was associated with a 25% increase in promoter strength, while a downstream BRE correlated with a 10% decreased promoter strength. Promoters containing the Y patch and Inr elements showed a more robust expression than those without (Jores et al., 2021).

Alternatively, natural broadly effective core promoters can be utilized to expand the synthetic promoter’s utility. The 54-base pair core promoter of *CaMV35S* efficiently initiates transcription in both monocots and dicots (Jores et al., 2021).

#### 5.2.4 Validating the synthetic promoter

Experimental transient expression systems such as agroinfiltration or protoplast transfection can provide an initial activity profile of the promoter, which can then be confirmed using stable transgenics (**Figure 3**). Alternatively, Artificial Intelligence (AI) shows promise in developing predictive models for gene expression. Recently, Vaishnav et al. (2022) developed a highly accurate convolutional neural network that predicts gene expression levels in yeast using a training dataset of 30 million synthetic promoters. Such predictive models reduce the burden of experimentation and can help design synthetic promoters with specific strengths. However, deep learning methods may have limitations when applied in organisms with highly complex regulatory mechanisms, as DNA residing millions of base pairs away can influence the gene expression levels (Wagner, 2022).

Computationally or manually designed synthetic promoters offer predictable expression levels (directly proportional to the strength of CREs) compared to native promoters in heterologous systems (Cai et al., 2020). However, the inadvertent introduction of unknown or uncharacterized transcription factor binding sites (TFBSs) undermines the predictability of synthetic promoters (Cai et al., 2020). Expanding current TFBS databases is imperative to achieve predictable gene expression levels in plants. **Figure 3** depicts the methodology discussed for designing and validating a synthetic promoter.

## 6 Transcription enhancers for synthetic promoters

TFs bound to enhancer sites recruit complexes that stimulate transcription (Panigrahi and O’Malley, 2021). Unlike promoters, enhancer elements function regardless of orientation and can be active over long distances (Panigrahi and O’Malley, 2021). Identifying enhancer-gene combinations is critical for targeted expression and tissue specificity. Enhancer regions in the genome can be identified by relying on characteristics such as nucleosome deficiency, DNAse hypersensitivity, bidirectional transcription, and occupancy by TF clusters, co-activators, and RNA polymerase II (Arner et al., 2015).

Experimental approaches employed to identify enhancers include i. Assessing global transcription potential by Global run-on sequencing (GRO-seq) or mapping transcription start sites by cap analysis (Jores et al., 2020); ii. Assessing transcription activation of potential enhancer regions using reporter genes (Jores et al., 2020); iii. Targeted suppression or activation of CREs; iv. Genome-wide assessment of chromatin connectomes (Arner et al., 2015). These approaches have been successfully employed in human and animal genomes to recognize potential enhancer regions (De Santa et al., 2010; Yamashita et al., 2011).

However, transcriptional regulation in plants varies widely from that of animals. Plant transcription is predominantly regulated at the level of initiation, and proximal promoter pausing is generally absent (Hetzel et al., 2016). Therefore, loose transcripts from enhancer sites are rarely produced, which rules out the possibility of identifying enhancer sequences by mapping nascent RNA transcripts (Hetzel et al., 2016). Self-transcribing active regulatory region sequencing (STARR-seq), a massively parallel reporter assay, identifies potential enhancers genome-wide and quantitates their strength by reporter expression (Arnold et al., 2013). STARR-seq has been performed in rice, maize, and tobacco (Jores et al., 2021; Ricci et al., 2019; Sun et al., 2019). A recently developed tobacco *in-planta* system for STARR-seq can detect stimuli-specific CREs (Jores et al., 2021).

Plant enhancer sequences increase the transcription strength of minimal promoters, but their activity depends on their location (upstream or downstream of the core promoter) and the plant species (Jores et al., 2021). Although some studies claim that plant genes contain transcription enhancer elements in the first intron, potent enhancers are generally absent in transcribed regions (Laxa et al., 2016; Samadder et al., 2008). In tobacco, enhancers were found to be inactive in the transcribed region but performed better when placed immediately upstream of the minimal promoter (Jores et al., 2020). When tested in tobacco leaves and maize protoplasts, the strong viral 35S enhancer increased transcription for almost all the tested *Arabidopsis*, maize, and sorghum core promoters (Jores et al., 2021). The enhancer responsiveness of core promoters positively correlated with expression specificity and the presence of the TATA box. However, proximal CREs that increased promoter strength in many cases decreased enhancer responsiveness, potentially owing to the limited pool of TFs or TF incompatibility (Jores et al., 2021).

Enhancer sequences are a dense cluster of TFBSs with binding motifs typically shorter than 10 base pairs (Panigrahi and O’Malley, 2021). An enhancer’s potential is determined by the type of bound TF and the number, order, orientation, binding affinity, and spacing of individual TFBS in the sequence (Panigrahi and O’Malley, 2021). Many experimental techniques, such as chromatin immunoprecipitation and sequencing, protein binding microarrays (Berger and Bulyk, 2009), systematic evolution of ligands by exponential enrichment and sequencing (Jolma et al., 2010), DNA affinity purification sequencing, chromatin immunoprecipitation-STARR-seq, have been developed to identify TFBS in a high through-put manner *in-vitro* or *in-vivo* using endogenous or recombinant TFs (Lai et al., 2019). Although these techniques are not without drawbacks, they have resulted in TFBS databases, including TRANSFAC (Matys et al., 2003), JASPAR (Khan et al., 2018), Universal PBM Resource for Oligonucleotide Binding Evaluation (UniPROBE) (Hume et al., 2015), Homo Sapiens Comprehensive Model Collection (HOCOMOCO) (Kulakovskiy et al., 2013), Catalog of Inferred Sequence Binding Preferences (CIS-BP) (Weirauch et al., 2014), and SwissRegulon (Pachkov et al., 2012).

Despite the progress achieved in TFBS mapping, designing an enhancer module with predictable gene regulatory effects is difficult (Slattery et al., 2014). TFBS have mostly conserved functions but can display sequence divergence (Slattery et al., 2014). Many TFs are indirectly recruited to the enhancer region through protein-protein interactions with bound TFs (Panigrahi and O’Malley, 2021). Several high-throughput studies have been conducted in yeast, *Drosophila melanogaster*, and human cells to understand the regulatory principles driving promoter activity (de Boer et al., 2020). However, large-scale analysis of plant regulatory elements is lacking. The role of regulatory elements in plants has mainly been studied in relation to animal data screening. Moreover, plant promoter sequences are much more extensive than other organisms (Jores et al., 2020). Our limited understanding of plant regulatory regions poses a significant challenge in designing synthetic modules for improved transgene expression.

## 7 Transcription factors for synthetic promoters

Efficient and specific TFs are critical for the functioning of engineered circuits. Using native TFs, especially in plants with complex intertwined regulatory mechanisms, can derail endogenous pathways (Andres et al., 2019). A suite of orthogonal artificial transcription factors and corresponding synthetic promoters can be used to achieve a range of predictable gene outputs. Incorporating specific and unique binding sites of synthetic TFs in promoters helps reduce potential off-target effects (Uniyal et al., 2019). Modulating the number and location of TFBSs also assists in fine-tuning gene expression strength (Rantasalo et al., 2018).

**Figure 5:**
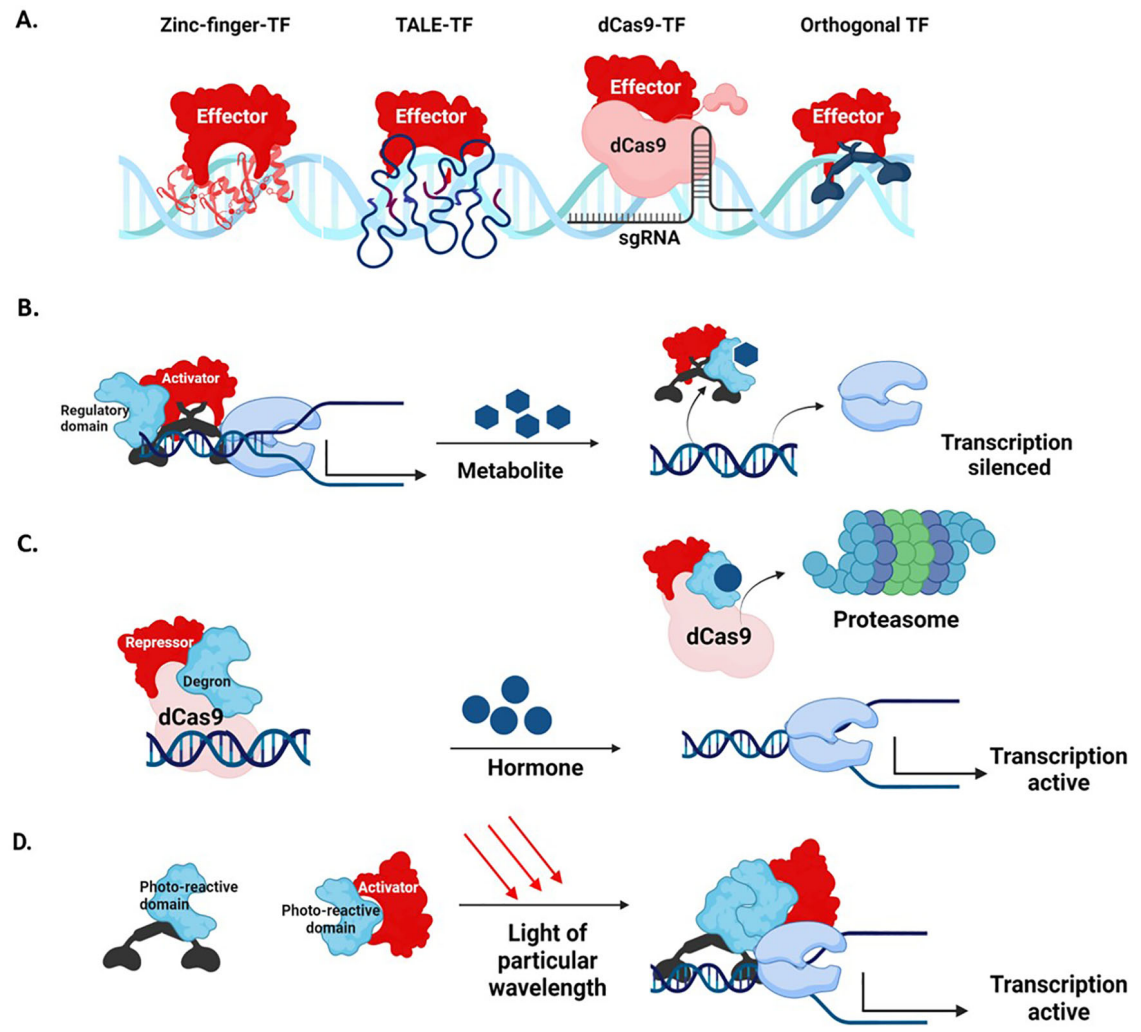
Synthetic transcription factors **(A)** Different types of DBDs bound to an effector domain **(B)** Regulation of a synthetic activator by a metabolite. On exposure, the activator binds to the metabolite and releases it from the transcription complex, silencing the target gene **(C)** Regulation of synthetic dCas9-repressor by a hormone-responsive degron sequence. When exposed to the hormone, the degron sequence directs the dCas9-repressor to the proteasome and de-represses the expression of the target gene **(D)** Optogenetic regulation. The DNA-binding and activator domains are fused to photo-reactive proteins that dimerize when exposed to light rays of particular wavelengths. On dimerization, the complete TF binds to the DNA and activates transcription.

Synthetic TFs are engineered by fusing tailored or natural orthogonal DNA-binding domains (DBDs) to effector domains (**Figure 5A**). Natural orthogonal DBDs, such as that of yeast Gal4, *Pseudomonas* PhlF, can be utilized by incorporating their binding sites into the promoter (Brophy et al., 2022). A library of synthetic transcriptional modules (including promoters, activators, and repressors) for plants has been created utilizing the orthogonal regulatory systems of *Saccharomyces* species (Belcher et al., 2020). Conversely, *Arabidopsis* TFs have been used to establish a library of orthogonal regulators in *Saccharomyces cerevisiae* (Naseri et al., 2017).

Tailored DBDs such as the Zinc Finger (ZF) domain, TALE domain, and the endonuclease dead Cas9 (dCas9) can target virtually any sequence (Kabadi and Gersbach, 2014). ZF protein domains contain a tandem array of cysteine and histidine (Cys2-His2) residues that detect a roughly 3-base pair DNA sequence (Bogdanove et al., 2018; Zetsche et al., 2015). DNA-binding by TALE is mediated by arrays of highly conserved 33-35 amino acid length repeats, with each repeat recognizing a single DNA base (Bogdanove et al., 2018; Zetsche et al., 2015). The dCas9 domain is recruited to the target by a sgRNA sequence. Through a cassette of sgRNAs, dCas9-TFs simultaneously control the expression of multiple promoters (Uniyal et al., 2019). Multiplex genome editing via dCas9-TFs allows simultaneous activation of multiple distinct genomic loci (Li et al., 2017; Lowder et al., 2018; Pan et al., 2021; Xiong et al., 2021). As amino acids do not define DNA binding, dCas9-TFs are comparatively easy to engineer (Lowder et al., 2015). The complex designing approach necessary for multiplex targeting is simplified and streamlined through a molecular toolbox of CRISPR Cas9 T-DNA constructs assembled through Golden Gate cloning (Lowder et al., 2015).

The effector domains of synthetic TFs are derived from natural TFs, such as the acidic VP16 activation domain from the herpes simplex virus or its derivative VP64 domain, the *Arabidopsis* Ethylene Response Factor 2 activation domain (ERF2AD), maize C1 domain, and EDLL (Li J. et al., 2013). The tobacco EAR and its derivative SRDX domains, Krueppel-Associated Box (KRAB), are repressors inhibiting transcription through chromatin remodeling (Mahfouz et al., 2012; Lupo et al., 2013). In recent years, large-scale characterization of effector domains from humans and yeast has been conducted (Tycko et al., 2020; Arnold et al., 2018). A similar study of *Arabidopsis* effector domains through *Agrobacterium*-mediated transient expression assay revealed 166 activator and 53 repressor domains. Of the activators, 49 displayed more potent activity than the VP16 domain, with the strongest cold response CBF4 activator giving a 16-fold increase in reporter expression compared to basal levels (Hummel et al., 2023).

In CRISPR-based transcription activation (CRISPRa), enhanced modulation can be achieved through effector domain stacking on a single DBD (Ding et al., 2022). In the dCas9 Super Nova Tag (SunTag) system, stacking is mediated through tandem General Control Nonderepressible4 (GCN4) peptide repeats, while in the scRNA system, stacking is mediated through a scaffold RNA. In SunTag and scRNA systems, the transcription regulators bind to dCas9 via anti-GCN4 antibodies and RNA-binding proteins, respectively (Morita et al., 2020; Zalatan et al., 2015). In the CRISPR ACT2.0 and ACT3.0 systems, the activator domains are recruited to a modified guide RNA (gRNA) that acts as a scaffold (Lowder et al., 2018; Pan et al., 2021). Alternatively, multiple effector domains are fused directly to the C-terminus of the dCas9 protein. The dCas9-TV system containing a combination of VP128 and six TALE activation domains has been applied in plant cells (Li et al., 2017; Xiong et al., 2021).

### 7.1 Regulation of synthetic TFs

Synthetic TFs can be regulated by placing them under a promoter offering conditional expression or by including regulatory domains in the TF. These regulatory domains may be responsive to a specific metabolite (Garagounis et al., 2021), hormone (Khakhar et al., 2018), or light of particular wavelengths (optogenetic regulation) (Shikata and Denninger, 2022). Synthetic TFs with metabolite-responsive regulatory domains can be utilized as intrinsic plant biosensors that couple plant metabolite production with reporter gene expression. (Khakhar et al., 2018) (**Figure 5B**). dCas9 repressors with hormone-responsive degron sequences activate the transcription of target genes when exposed to the hormone (Khakhar et al., 2018) (**Figure 5C**). Optogenetic switches use light-sensitive dimerizing proteins fused to DBD and effector domains. In response to light, the regulatory domains dimerize and control the transcription of target genes (Shikata and Denninger, 2022) (**Figure 5D**).

Optogenetic switches are challenging to implement in plants as light is indispensable for growth. Plant useable light switch element (PULSE) overcomes this constraint by using constitutively expressed dual switches of blue and red light-responsive photoreceptors fused to a repressor and activator domain, respectively. Under white light, the repressor overcomes activation to suppress gene expression. Under monochromatic red light alone, the activator initiates gene transcription (Ochoa-Fernandez et al., 2020).

Synthetic ZF-TFs and TALE-TFs have been used to activate or suppress endogenous and reporter genes in *Arabidopsis*, maize, rice, tobacco, *Brassica napus*, and *Chlamydomonas reinhardtii* (Morbitzer et al., 2010; Li T. et al., 2013; Tol et al., 2019). Synthetic hormone-responsive dCas9-based repressors that respond to exogenous hormone treatment and differences in endogenous hormone levels have been developed. These can be leveraged to study endogenous hormone distributions and reprogram plant signals to influence plant morphology. Auxin-responsive dCas9 repressors targeting the auxin transporter PIN1 suppressed the gene’s transcriptional positive feedback mechanism and reduced lateral branching (Khakhar et al., 2018). A reprogrammable CRISPR-dCas9-dependent bipartite unit (consisting of a dCas9 fused to an activator and multiple sgRNAs targeting different genes) is proposed to activate several genes simultaneously (Shrestha et al., 2018). Under the influence of a stress-inducible synthetic promoter, the dCas9-activator is expressed along with a transcript of multiple short guide RNAs. The short guide RNAs will define the target promoters of the activator and, in a positive-feedback mechanism, upregulate its transcription (Shrestha et al., 2018). **Table 2** lists the different synthetic transcription factors developed and utilized for regulating endogenous genes or transgenes in plants.

**Table 2:** Synthetic transcription factors utilized in plant systems.

DNA-binding domain	Effector domain	Effector function	Application	Reference
Zinc-finger domain	VP16 VP64	Activator	5-7-fold increase in endogenous *At4CL1* transcript levels in *Arabidopsis thaliana* under constitutive promoter	Sánchez et al., 2006; Gupta et al., 2012
	C1	Activator	Heritable 20-fold increase in α-tocopherol percentage in seed under seed-specific promoter	Van Eenennaam et al., 2004
			Plant-based zinc-finger domain under CaMV35S constitutive promoter showed 5-25-fold increase in *At*ADH protein activity	Holmes-Davis et al., 2005
	CRP	Activator	Chloroplast-targeted zin-finger activator under *AtFEDS* promoter showed variable photosynthetic performance and growth depending on T-DNA insertion site	Tol et al., 2019
	LuxR	Activator	Less-effective chloroplast-targeted zinc-finger activator due to residual DNA-binding activity of LuxR effector domain	
	KRAB	Repressor	15-20-fold decrease in endogenous *At4CL1* transcript levels in *Arabidopsis thaliana*	Sánchez et al., 2006
TALE	XcvAvrBs3; XcvAvrBs4	Activator	Increased transcript levels of *AtEGL3* and *AtKNAT1* induced by dTALE[*EGL3*] and dTALE[*KNAT1*], respectively, under CaMV35S promoter	Morbitzer et al., 2010
			A library of dTALE activated synthetic promoters exhibiting expression levels varying from 5-90% of that of CamV35S promoter	Brückner et al., 2015
	XooAvrXa10	Activator	dTALE-xa27 demonstrated as an active avirulence factor against *Xanthomonas oryzae* pv *oryzae* corresponding to the disease-resistant gene *xa27* in rice cultivar IR64	Li T. et al., 2013
Mutiplexed TALE	VP64	Activator	Simultaneous targeted activation of three *Arabidopsis* genes *CSTF64, GL1, RBP-DR1*	Lowder et al., 2018
Gal4	VP16 TAC3d2	Activator	GAL4:TAC3d2 and GAL4:VP16 binding to the hybrid promoter FM’M induced high levels of recombinant protein production. GAL4:TAC3d2 activation induced greater effect than GAL4:VP16 as TAC3 activates transcription at both proximal and distal locations of the promoter.	Yun et al., 2023
Yeast DBD (MCM1, Gat1, Mata1, Matα2, Yap1)	VP16 C1	Activator	A library of synthetic TFs designed by leveraging orthogonal regulatory systems from *Saccharomyces* spp.	Belcher et al., 2020
	STDX	Repressor		
Prokaryotic DBD (TetR, LacI, LexA, PhlF)	VP16 ERF2	Activator	A library of synthetic TFs designed by leveraging orthogonal bacterial regulatory systems. Nine out of ten synthetic activators induced 3-45-fold increase in transcription.	Brophy et al., 2022
	TOPLESS(TPL) ZAT10(Z10) ERF4	Repressor	A library of synthetic TFs designed by leveraging orthogonal bacterial regulatory systems. Only four of the tested synthetic repressors generated more than 2 factor change in gene expression. None of the tested repressors achieved complete gene repression.	Brophy et al., 2022
dCas9	VP64	Activator	Orthogonal control system with dCas9:VP64 activator and gRNA expression cassettes under inducible POLII promoters	Kar et al., 2022
			2-7-fold increase in transcriptional activation of *AtPAP1* and 3-7.5-fold increase in transcriptional activation of *AtmiR319* by dCas9:VP64 in transgenic *Arabidopsis.* The increased expression failed to generate overexpression phenotype in *Arabidopsis*.	Lowder et al., 2015
			1.6-5.6-fold increase in transcriptional activation of *UFGT* in transgenic grape	Ren et al., 2022
	EDLL TAL	Activator	dCas9:EDLL and dCas9:TAD mediated 5-fold and 6-fold increase in transcriptional activation in transient assay	Piatek et al., 2015
	TV	Activator	Endogenous *AtWRKY30* and *RLP23* transcriptional activation by 48-139-fold and over 30-fold in *Arabidopsis*	Li et al., 2017
			Endogenous *OsGW7* and *OsER1* transcriptional activation by up to 3,738-fold in rice, persisting at least till the 4^th^ generation	Xiong et al., 2021
			5.7-7.2-fold increase in transcriptional activation of *UFGT* in grape	Ren et al., 2022
	SunTag	Activator	dCas9:VP64 with additional p65-HSF generated the overexpression phenotype of *AtPAP1* in *Arabidopsis*	Park et al., 2017
			SunTagVP64 is highly specific and efficient for multiplexing compared to zinc finger activators or TAL effectors	Papikian et al., 2019
	VP64-EDLL	Activator	Tissue-specific expression of dCas9:VP64 EDLL and constitutive expression of gRNA cassette resulted in ~2-5-fold increase in endogenous CGA1 levels depending on site of transgene integration	Lee et al., 2021
	CRISPR ACT2.0	Activator	CRISPR-ACT2.0 induced 30-45-fold activation of *PAP1* and up to 1500-fold activation of *FIS2* in *Arabidopsis*, a 3-4-fold improvement over dCas9-VP64	Lowder et al., 2018
	CRISPR ACT3.0	Activator	CRISPR-ACT3.0 simultaneously targeted *AtFT* and *AtTCL1* and induced 130-fold-240-fold and 3-8-fold activation, respectively, in *Arabidopsis*.	Pan et al., 2021
	SRDX	Repressor	dCas9:SRDX-mediated significant repression of *phytoene desaturase* in *Nicotiana benthamiana* through gRNA-guided binding to the sense strand of the promoter and the first exon	Piatek et al., 2015
	Degron sequence+TOPLESS (TPL)	Repressor	Hormone-sensitive dCas9 repressors used as sensors to endogenous hormone distribution; auxin-sensitive dCas9 repressor suppressed auxin transporter PIN1 expression and altered shoot architecture in *Arabidopsis*	Khakhar et al., 2018

Several studies in plants have demonstrated the feasibility of synthetic transcription factors in driving *in vivo* gene expression. However, when selecting appropriate DNA binding and effector domains, the desired strength and specificity of gene expression must be considered. Lowder et al., 2018 proposed the existence of a fold-activation ceiling for endogenous genes; above this threshold, gene silencing mechanisms are triggered. Genes with very low baseline expression achieved higher fold activation through synthetic TFs, but increasing the expression of genes under vigorous transcription resulted in transcriptional repression or gene silencing. Furthermore, different types of synthetic activators can exhibit varying activation efficiencies for the same gene. Li et al. (2017) found that dCas9:VP64 and dCas9:TV TFs displayed contrasting activation efficiencies for the endogenous gene *RLP23*. While dCas9:TV induced a 44-fold activation, dCas9:VP64 slightly suppressed *RLP23* expression in *Arabidopsis*.

Synthetic DBDs with unique binding sites maximize target specificity. However, synthetic DBDs can still display off-target binding to homologous sequences. Online prediction tools can assist researchers in designing unique sgRNAs with reduced off-target effects (Guo et al., 2023). Alternatively, a recent study in yeast has highlighted the advantage of weakly binding cooperative TF assemblies over lock-and-key specificity. Bragdon et al., 2023 found that high specificity emerged from cooperative interactions of TFs that are individually weak and non-specific. The cooperative TF assemblies enhanced circuit performance and reduced circuit-imposed fitness cost, thereby increasing the evolutionary stability of synthetic circuits.

## 8 The success of synthetic genetic circuits in plants

Plant regulatory mechanisms sense and integrate various exogenous (environmental) and endogenous (genetic, developmental, metabolic) cues (Andres et al., 2019). Most plant signaling pathways comprise multiple components, exhibit extensive feedback control, cross-interaction with other networks, and redundancy of function (Pokhilko et al., 2013; Fogelmark and Troein, 2014). Therefore, targeted manipulation of native signaling circuits is challenging. Novel networks can substitute a plant’s native metabolic routes and systems. Fully synthetic circuits with orthogonal sensing, signaling, and output elements offer the advantage of minimizing cross-talk with endogenous pathways. They can improve the efficiency of signaling cascades by bypassing endogenous regulation (Andres et al., 2019).

Synthetic genetic circuits aim to introduce novel functionalities in plants including improving crop productivity and the production of pharmaceuticals. There exist different types of synthetic circuits. Simple circuits integrate a few signals to perform basic functions. Toggle circuits generate two switchable outputs arranged in feedback such that one controls the other, and self-sustaining oscillatory circuits are characterized by periodic expression. Boolean logic gate circuits that combine multiple input signals to give a defined yes-or-no output have been designed and implemented in prokaryotic, yeast and animal models (Andres et al., 2019; Baldwin et al., 2016).

However, introducing synthetic circuits in plants is an exhaustive design-build-test-learn cycle. A robust synthetic circuit must be able to tolerate cellular perturbations and function seamlessly along with endogenous pathways (Liu et al., 2013). A specifications-based synthetic systems biology approach involving *in silico* model-based computer simulations and mathematical analysis allow several designs to be tested and optimized before committing to *in-vitro* or *in-vivo* realization (Baldwin et al., 2016). Ideally, repositories of biological models, curated circuit parts, and associated parameter values can simplify circuit design and optimization. However, such databases are still in their infancy, especially for plant systems. In the past decade, several computational tools have been created for designing and behavior prediction of synthetic circuits (Devarajan et al., 2022). Although most of these tools are optimized for prokaryotic systems, they can be adopted for plants, provided orthogonal parts are available (Devarajan et al., 2022). Systems and synthetic biology approaches can feed into each other to build on complex genetic networks (Chen and Wu, 2014).

Tuning and optimization of circuit and circuit parts require quantitative assays that can assess functioning in a short duration and high throughput manner. Brophy et al., 2022 analyzed the gene expression and specificity of a collection of synthetic transcriptional regulators and logic gate circuits through *Agrobacterium*-mediated expression assay in *Nicotiana benthamiana*. The synthetic regulators drove the expression of the green fluorescent protein, which was normalized against a constitutively expressed mCherry encoded on the same T-DNA. A dual luciferase (*Renilla*: firefly) reporter assay has also been developed to test circuits and circuit components in *Arabidopsis* protoplasts (Khan et al., 2024).

Several Boolean logic circuit designs have been tested in plants in recent years. Brophy et al., 2022 designed a collection of logic gates and tested their capacity to give specific spatial expression patterns in *Arabidopsis* root tissues. The designed BUFFER gates were applied to express *slr-1*(mutant *INDOLE-3-ACETIC ACID INDUCIBLE14* regulator that eliminates root branching) in lateral root stem cells. *slr-1* expression was activated through the AmtR-VP16 TF placed under *GATA23* (a lateral root-specific promoter). The synthetic promoter driving *slr-1* contained multiple operator sites mutated to give a range of expressions that translated into plants showing a gradient of root densities. Khan et al. (2024) have designed and tested a collection of CRISPR interference (CRISPRi)-based logic circuits in *Arabidopsis* protoplasts.

Complex circuits can be created with logic gates feeding into each other to achieve high levels of transcriptional control and insulation from endogenous pathways. Orthogonal TF- and CRISPRi-based circuits depend on input signals to induce transient changes in gene expression. Recombinase-based memory gene circuits offer an output activity that persists beyond the input signal and locks in the expression state (Lloyd et al., 2022). In recombinase-based circuits, the system is in the ‘OFF’ state until exposed to the input signal. The ‘OFF’ state is achieved by introducing a strong transcriptional terminator, flanked by recombination sites, between the gene’s promoter and coding region. When exposed to the input signal, the recombinase enzyme cleaves the terminator sequence turning the system ‘ON’ (Lloyd et al., 2022). The target gene can be repressed by flanking the promoter or the coding region with recombinase sites. The input signal, or the recombinase enzyme’s conditional expression, is executed by placing it under an inducible promoter. Lloyd et al., 2022 designed recombinase-based 1 and 2- input logic gates with Flippase (Flp) and B3 recombinases and demonstrated their functioning in *Arabidopsis*.

Synthetic circuits require rigorous tuning. Well-characterized circuit components may not function predictably in the system (Brophy et al., 2022). The type and architecture of the construct also affect gene expression levels (Khan et al., 2024; Lloyd et al., 2022). Multiple-switch circuits consider many input signals to define output and can reduce leaky expression but are hard to stabilize. An imbalanced expression of inputs can break down the circuit, especially in circuits involving TFs. A leaky or aberrant gene expression is intolerable for many applications.

Stable transgenes often experience gene-silencing effects (Rajeevkumar et al., 2015). In the context of a synthetic circuit, diminished expression of a single transgene can interfere with the whole circuit operation, making it imperative to minimize gene silencing triggers. Gene silencing can occur through sequence-dependent inactivation by acquiring specific epigenetic markers (Bewick and Schmitz, 2017). A comparative study in *Arabidopsis* found striking epigenetic differences in endogenous genes and transgenes, owing to the difference in reactivity to the H3K4me3 demethylase, JMJ14 (Butel et al., 2021).

Promoters differ in sensitivity to epigenetic silencing (Cabrera et al., 2022). However, as silencing effects also depend on the locus of integration and cell type, it is difficult to ascertain the effect of the promoter alone. The promoter’s influence on gene silencing can be tested by controlling for genomic context and varying the selected promoter (Cabrera et al., 2022). Performing such characterizations on a large scale will assist in promoter selection.

In plants, RNA-dependent RNA polymerases initiate post-transcriptional gene silencing (PTGS) by dsRNA formation from aberrant RNA (Zhang et al., 2015). RNA Quality Control (RQC) pathways check aberrant RNA produced from endogenous genes and transgenes exceeding a certain threshold. However, high levels of transgene expression can result in RQC failure and RDR-dependent PTGS (Martínez de Alba et al., 2015). Transgenes expressed under a strong promoter and/or tandem copies of transgenes induce PTGS most efficiently (Sidorenko et al., 2017). As synthetic promoters are designed to offer strong activity, they are susceptible to silencing effects. Employing inducible systems with external control (through small molecule/stress-/light-stimulus) to maintain a tightly regulated OFF state provides an additional layer of safety (Cabrera et al., 2022). The orthogonality of circuit components also reduces off-target effects and homology-dependent gene silencing (Yasmeen et al., 2023). Expanding the toolkit of orthogonal promoters and TFs for plants will provide greater flexibility to circuit design and insulation from endogenous pathways.

## 9 Conclusion

The agricultural challenges to be faced may require the advanced solution of synthetic biology. Multigene circuits aim to engineer plants with novel functionalities. Such complex circuits need tight transcriptional control with minimal cross-talk and homology with endogenous sequences. Promoters are vital elements in circuits that decide the spatiotemporal expression of genes. The order, copy number, and spacer length of CREs are critical factors that determine the strength and activity of the promoter. Decades of research have identified CREs that act in particular stimuli. However, databases of CREs are still incomplete, as many plant regulatory mechanisms remain unclear. Combining synthetic promoters with synthetic enhancers and TFs ensures better regulation of multigene circuits. Genome-wide studies of plant enhancer regions are lacking, causing TFBS databases to remain incomplete. TFBS redundancy, indirect recruitment of unknown TFs, and insufficient knowledge of plant enhancers are responsible for the difficulty in designing enhancer modules with predictable strength and function. Large-scale genome-wide studies in different plant models are needed to understand better and apply their complex regulatory mechanisms.

Expanding the repertoire of synthetic regulatory modules to include a variety of stimuli- and tissue-specific promoters, enhancers, silencers, activators, repressors, and TFs is necessary to achieve fine-tuned circuits with specific spatiotemporal expression patterns. Toolkits of synthetic promoters, activators, and TFs have been designed for plant use. Despite the progress in designing synthetic regulatory elements, establishing entire circuits with multiple transgenes and separate regulatory networks is a challenge that requires repeated design-build-test cycles. Characterized circuit elements can behave unpredictably when introduced into plant systems. Synthetic circuits can impact the intricate regulatory mechanisms that vary among cells. Transient expression methods and the advances in single-cell measurements and spatially resolved omics can potentially provide more precise measurements of circuit strength and further develop circuit technology. Strategies like single-cell RNA sequencing can help discover cell type-specific promoters, CREs, and miRNAs that enable cell-specific gene expression and minimize pleotropic effects (Marand et al., 2021; Zhang et al., 2019). However, the broad use of single-cell omics approaches faces several challenges including the lack of standardized protocols for sample preparation and data analysis across plant species (Islam et al., 2024). Efforts in this direction will increase reproducibility and expand the omics databases. Integrating different omics data can enhance the researcher’s comprehension of cellular processes. Integrative analysis platforms like GLUE (graph-linked unified embedding) (Cao and Gao, 2022) and Con-AAE (Contrastive cycle adversarial Autoencoders) (Wang et al., 2023) need to be further developed to handle the vast amount of data collected across plant species.

## Glossary

ABRE: Abscisic Acid-Responsive Element
BRE: B Recognition Element
CaMV35S: Cauliflower Mosaic Virus 35S
CE: Coupling Element
CIS-BP: Catalog of Inferred Sequence Binding Preferences
CLE: CLAVATA3/ESR-Related
csLOB1: *Citrus sinensis* Lateral Organ Boundary 1
CoMoVa: Conservation of Motif Variants
CRE: Cis-Regulatory Element
CRISPR/Cas9: Clustered Regularly Interspaced Short Palindromic Repeats/CRISPR-Associated protein
CRISPRi: CRISPR interference
DBD: DNA Binding Domain
DGAT2: Diacylglycerol Acyltransferase 2
DRE: Dehydration-Responsive Element
DREB/CBF: Dehydration Responsive Element Binding factors/C repeat element Binding Factors
ERF2^AD^: Ethylene Response Factor 2 Activation Domain
Flp: Flippase
GCN4: General Control Nondepressible
gRNA: guide RNA
HOCOMOCO: Homo Sapiens Comprehensive Model Collection
HOMER: Hypergeometric Optimization of Motif EnRichment
Inr: Initiator
KRAB: Krueppel-associated box
MEME: Multiple Expectation maximizations for Motif Elicitation
MCOT: Motifs Co-Occurrence Tool
PLACE: Plant cis-acting regulatory DNA elements
Plant CARE: Plant cis-acting regulatory elements, enhancers and repressors
PlantRegMap: Plant Transcriptional Regulatory Map
scRNA: scaffold RNA
sgRNA: short guide RNA
SpaMo: Spaced Motif Analysis
SPECS: Synthetic Promoters with Enhanced Cell-State Specificity
STARR-seq: Self-Transcribing Active Regulatory Region-sequencing
SunTag: Super Nova Tag
TALEN: Transcription Activator-Like Effector Nuclease
TEF1: Translation Elongation Factor 1
TF: Transcription Factor
TFBS: Transcription Factor Binding Site
TRANSFAC: Transcription Factor database
TCTP: Translationally Controlled Tumor Protein
TSS: Transcription Start Site
UniPROBE: Universal PBM Resource for Oligonucleotide Binding Evaluation
VP-16/64: Viral Protein 16/64
ZFN: Zinc-Finger Nuclease

## References

[B1] Acharya S. Sengupta S. Patro S. Purohit S. Samal S. K. Maiti I. B.. (2014). Development of an intra-molecularly shuffled efficient chimeric plant promoter from plant infecting Mirabilis mosaic virus promoter sequence. J. Biotech. 169, 103–111. doi: 10.1016/j.jbiotec.2013.08.022 24060830

[B2] Andres J. Blomeier T. Zurbriggen M. D. (2019). Synthetic switches and regulatory circuits in plants. Plant Physiol. 179, 862–884. doi: 10.1104/pp.18.01362 30692218 PMC6393786

[B3] Arner E. Daub C. O. Vitting-Seerup K. Andersson R. Lilje B. Drabløs F.. (2015). Transcribed enhancers lead waves of coordinated transcription in transitioning mammalian cells. Science 347, 1010–1014. doi: 10.1126/science.1259418 25678556 PMC4681433

[B4] Arnold C. D. Gerlach D. Stelzer C. Boryń Ł.M. Rath M. Stark A. (2013). Genome-wide quantitative enhancer activity maps identified by STARR-seq. Science 339, 1074–1077. doi: 10.1126/science.1232542 23328393

[B5] Arnold C. D. Nemčko F. Woodfin A. R. Wienerroither S. Vlasova A. Schleiffer A.. (2018). A high-throughput method to identify trans-activation domains within transcription factor sequences. EMBO J. 37, e98896. doi: 10.15252/embj.201798896 30006452 PMC6092621

[B6] Aysha J. Noman M. Wang F. Liu W. Zhou Y. Li H.. (2018). Synthetic promoters: designing the cis regulatory modules for controlled gene expression. Mol. Biotechnol. 60, 608–620. doi: 10.1007/s12033-018-0089-0 29855997

[B7] Bai J. Wang X. Wu H. Ling F. Zhao Y. Lin Y.. (2020). Comprehensive construction strategy of bidirectional green tissue-specific synthetic promoters. Plant Biotechnol. J. 18, 668–678. doi: 10.1111/pbi.13231 31393049 PMC7004895

[B8] Baldwin G. Bayer T. Dickinson R. Ellis T. Freemont P. S. Kitney R. I.. (2016). Synthetic biology: A primer (UK: Imperial College Press). doi: 10.1142/p1060

[B9] Belcher M. S. Vuu K. M. Zhou A. Mansoori N. Agosto Ramos A. Thompson M. G.. (2020). Design of orthogonal regulatory systems for modulating gene expression in plants. Nat. Chem. Biol. 16, 857–865. doi: 10.1038/s41589-020-0547-4 32424304

[B10] Berger M. F. Bulyk M. L. (2009). Universal protein-binding microarrays for the comprehensive characterization of the DNA-binding specificities of transcription factors. Nat. Protoc. 4, 393–411. doi: 10.1038/nprot.2008.195 19265799 PMC2908410

[B11] Bewick A. J. Schmitz R. J. (2017). Gene body DNA methylation in plants. Curr. Opin. Plant Biol. 36, 103–110. doi: 10.1016/j.pbi.2016.12.007 28258985 PMC5413422

[B12] Bhadouriya S. L. Karamchandani A. N. Nayak N. Mehrotra S. Mehrotra R. (2024). Artificially designed synthetic promoter for a high level of salt induction using a cis-engineering approach. Sci. Rep. 14, 13657. doi: 10.1038/s41598-024-64537-z 38871942 PMC11176371

[B13] Bhadouriya S. L. Suresh A. Gupta H. Mehrotra S. Gupta D. Mehrotra R. (2021). In Silico Analysis of CCGAC and CATGTG Cis-regulatory Elements Across Genomes Reveals their Roles in Gene Regulation under Stress. Curr. Genom. 22, 353. doi: 10.2174/2F1389202922666210601095700 PMC8844943 35283666

[B14] Bhalothia P. Alok A. Mehrotra S. Mehrotra R. (2013). AACA element negatively interferes with expression of Protein Phosphatase 2C like gene promoter. Am. J. Plant Sci. 4, 549–554. doi: 10.4236/ajps.2013.43071

[B15] Bhatnagar-Mathur P. Devi M. J. Reddy D. S. Lavanya M. Vadez V. Serraj R.. (2007). Stress-inducible expression of At DREB1A in transgenic peanut (*Arachis hypogaea L.*) increases transpiration efficiency under water-limiting conditions. Plant Cell Rep. 26, 2071–2082. doi: 10.1007/s00299-007-0406-8 17653723

[B16] Bhattacharjee S. Renganaath K. Mehrotra R. Mehrotra S. (2013). Combinatorial control of gene expression. BioMed. Res. Int. 2013, 407263. doi: 10.1155/2013/407263 24069600 PMC3771257

[B17] Bhullar S. Datta S. Advani S. Chakravarthy S. Gautam T. Pental D.. (2007). Functional analysis of cauliflower mosaic virus 35S promoter: re-evaluation of the role of subdomains B5, B4 and B2 in promoter activity. Plant Biotechnol. J. 5, 696–708. doi: 10.1111/j.1467-7652.2007.00274.x 17608668

[B18] Bhunia R. K. Menard G. N. Eastmond P. J. (2022). A native promoter–gene fusion created by CRISPR/Cas9-mediated genomic deletion offers a transgene-free method to drive oil accumulation in leaves. FEBS Lett. 596, 1865–1870. doi: 10.1002/1873-3468.14365 35490366 PMC9545981

[B19] Biłas R. Szafran K. Hnatuszko-Konka K. Kononowicz A. K. (2016). Cis-regulatory elements used to control gene expression in plants. (PCTOC) 127, 269–287. doi: 10.1007/s11240-016-1057-7

[B20] Bogdanove A. J. Bohm A. Miller J. C. Morgan R. D. Stoddard B. L. (2018). Engineering altered protein–DNA recognition specificity. Nucleic Acids Res. 46, 4845–4871. doi: 10.1093/nar/gky289 29718463 PMC6007267

[B21] Boni R. Chauhan H. Hensel G. Roulin A. Sucher J. Kumlehn J.. (2018). Pathogen-inducible Ta-Lr34res expression in heterologous barley confers disease resistance without negative pleiotropic effects. Plant Biotechnol. J. 16, 245–253. doi: 10.1111/pbi.12765 28561994 PMC5785347

[B22] Bragdon M. D. Patel N. Chuang J. Levien E. Bashor C. J. Khalil A. S. (2023). Cooperative assembly confers regulatory specificity and long-term genetic circuit stability. Cell 186, 3810–3825. doi: 10.1016/j.cell.2023.07.012 37552983 PMC10528910

[B23] Brophy J. A. Magallon K. J. Duan L. Zhong V. Ramachandran P. Kniazev K.. (2022). Synthetic genetic circuits as a means of reprogramming plant roots. Science 377, 747–751. doi: 10.1126/science.abo4326 35951698

[B24] Brückner K. Schäfer P. Weber E. Grützner R. Marillonnet S. Tissier A. (2015). A library of synthetic transcription activator-like effector-activated promoters for coordinated orthogonal gene expression in plants. Plant J. 82, 707–716. doi: 10.1111/tpj.12843 25846505 PMC4691316

[B25] Butel N. Yu A. Le Masson I. Borges F. Elmayan T. Taochy C.. (2021). Contrasting epigenetic control of transgenes and endogenous genes promotes post-transcriptional transgene silencing in Arabidopsis. Nat. Commun. 12, 2787. doi: 10.1038/s41467-021-22995-3 33986281 PMC8119426

[B26] Cabrera A. Edelstein H. I. Glykofrydis F. Love K. S. Palacios S. Tycko J.. (2022). The sound of silence: Transgene silencing in mammalian cell engineering. Cell Syst. 13, 950–973. doi: 10.1016/j.cels.2022.11.005 36549273 PMC9880859

[B27] Cai Y. M. Kallam K. Tidd H. Gendarini G. Salzman A. Patron N. J. (2020). Rational design of minimal synthetic promoters for plants. Nucleic Acids Res. 48, 11845–11856. doi: 10.1093/nar/gkaa682 32856047 PMC7708054

[B28] Cao Z. J. Gao G. (2022). Multi-omics single-cell data integration and regulatory inference with graph-linked embedding. Nat. Biotechnol. 40, 1458–1466. doi: 10.1038/s41587-022-01284-4 35501393 PMC9546775

[B29] Chen B. S. Wu C. C. (2014). Systems Biology: An Integrated Platform for Bioinformatics, Systems Synthetic Biology and Systems Metabolic Engineering (New York: Nova Publishers). 10.3390/cells2040635 PMC3972654 24709875

[B30] Ciolkowski I. Wanke D. Birkenbihl R. P. Somssich I. E. (2008). Studies on DNA-binding selectivity of WRKY transcription factors lend structural clues into WRKY-domain function. Plant Mol. Biol. 68, 81–92. doi: 10.1007/s11103-008-9353-1 18523729 PMC2493524

[B31] de Boer C. G. Vaishnav E. D. Sadeh R. Abeyta E. L. Friedman N. Regev A. (2020). Deciphering eukaryotic gene-regulatory logic with 100 million random promoters. Nat. Biotechnol. 38, 56–65. doi: 10.1038/s41587-019-0315-8 31792407 PMC6954276

[B32] De Santa F. Barozzi I. Mietton F. Ghisletti S. Polletti S. Tusi B. K.. (2010). A large fraction of extragenic RNA pol II transcription sites overlap enhancers. PloS Biol. 8, e1000384. doi: 10.1371/journal.pbio.1000384 20485488 PMC2867938

[B33] Devarajan A. Gupta D. Mitra K. Deb S. S. Reshamwala S. M. (2022). “Computational tools for design of synthetic genetic circuits,” in New Frontiers and Applications of Synthetic Biology (Academic Press), (159–169). doi: 10.1016/B978-0-12-824469-2.00015-4

[B34] Dhatterwal P. Mehrotra S. Miller A. J. Mehrotra R. (2021). Promoter profiling of Arabidopsis amino acid transporters: clues for improving crops. Plant Mol. Biol. 107, 1–25. doi: 10.1007/s11103-021-01193-1 34674117

[B35] Ding X. Yu L. Chen L. Li Y. Zhang J. Sheng H.. (2022). Recent progress and future prospect of CRISPR/Cas-derived transcription activation (CRISPRa) system in plants. Cells 11, 3045. doi: 10.3390/cells11193045 36231007 PMC9564188

[B36] Eraslan G. Avsec Ž. Gagneur J. Theis F. J. (2019). Deep learning: new computational modelling techniques for genomics. Nat. Rev. Genet. 20, 389–403. doi: 10.1038/s41576-019-0122-6 30971806

[B37] Fogelmark K. Troein C. (2014). Rethinking transcriptional activation in the Arabidopsis circadian clock. PloS Comput. Biol. 10, e1003705. doi: 10.1371/journal.pcbi.1003705 25033214 PMC4102396

[B38] Garagounis C. Delkis N. Papadopoulou K. K. (2021). Unraveling the roles of plant specialized metabolites: using synthetic biology to design molecular biosensors. New Phytol. 231, 1338–1352. doi: 10.1111/nph.17470 33997999

[B39] Ghoshdastidar D. Bansal M. (2022). Flexibility of flanking DNA is a key determinant of transcription factor affinity for the core motif. Biophys. J. 121, 3987–4000. doi: 10.1016/j.bpj.2022.08.015 35978548 PMC9674967

[B40] Guo C. Ma X. Gao F. Guo Y. (2023). Off-target effects in CRISPR/Cas9 gene editing. Front. Bioeng. Biotechnol. 11. doi: 10.3389/fbioe.2023.1143157 PMC10034092 36970624

[B41] Gupta M. DeKelver R. C. Palta A. Clifford C. Gopalan S. Miller J. C.. (2012). Transcriptional activation of Brassica napus β-ketoacyl-ACP synthase II with an engineered zinc finger protein transcription factor. Plant Biotechnol. J. 10, 783–791. doi: 10.1111/j.1467-7652.2012.00695.x 22520333

[B42] Gupta D. Dey N. Leelavathi S. Ranjan R. (2021). Development of efficient synthetic promoters derived from pararetrovirus suitable for translational research. Planta 253, 1–15. doi: 10.1007/s00425-021-03565-9 33475866

[B43] Gustin K. Burk R. D. (2000). PCR-directed linker scanning mutagenesis. Transcription factor Protoc. 130, 85–90. doi: 10.1385/1-59259-686-X:85 10589423

[B44] Heinz S. Benner C. Spann N. Bertolino E. Lin Y. C. Laslo P.. (2010). Simple combinations of lineage-determining transcription factors prime cis-regulatory elements required for macrophage and B cell identities. Mol. Cell 38, 576–589. doi: 10.1016/j.molcel.2010.05.004 20513432 PMC2898526

[B45] Hetzel J. Duttke S. H. Benner C. Chory J. (2016). Nascent RNA sequencing reveals distinct features in plant transcription. PNAS 113, 12316–12321. doi: 10.1073/pnas.1603217113 27729530 PMC5087027

[B46] Higo K. Ugawa Y. Iwamoto M. Korenaga T. (1999). Plant cis-acting regulatory DNA elements (PLACE) database: 1999. Nucleic Acids Res. 27, 297–300. doi: 10.1093/nar/27.1.297 9847208 PMC148163

[B47] Hume M. A. Barrera L. A. Gisselbrecht S. S. Bulyk M. L. (2015). UniPROBE, update 2015: new tools and content for the online database of protein-binding microarray data on protein–DNA interactions. Nucleic Acids Res. 43, D117–D122. doi: 10.1093/nar/gku1045 25378322 PMC4383892

[B48] Hummel N. F. Zhou A. Li B. Markel K. Ornelas I. J. Shih P. M. (2023). The trans-regulatory landscape of gene networks in plants. Cell Syst. 14, 501–511. doi: 10.1016/j.cels.2023.05.002 37348464

[B49] Islam M. T. Liu Y. Hassan M. M. Abraham P. E. Merlet J. Townsend A.. (2024). Advances in the application of single-cell transcriptomics in plant systems and synthetic biology. BioDesign Res. 6, 29. doi: 10.34133/bdr.0029 PMC10905259 38435807

[B50] Jameel A. Noman M. Liu W. Ahmad N. Wang F. Li X.. (2020). Tinkering cis motifs jigsaw puzzle led to root-specific drought-inducible novel synthetic promoters. Int. J. Mol. Sci. 21, 1357. doi: 10.3390/ijms21041357 32085397 PMC7072871

[B51] Jolma A. Kivioja T. Toivonen J. Cheng L. Wei G. Enge M.. (2010). Multiplexed massively parallel SELEX for characterization of human transcription factor binding specificities. Genome Res. 20, 861–873. doi: 10.1101/gr.100552.109 20378718 PMC2877582

[B52] Jores T. Tonnies J. Dorrity M. W. Cuperus J. T. Fields S. Queitsch C. (2020). Identification of plant enhancers and their constituent elements by STARR-seq in tobacco leaves. Plant Cell 32, 2120–2131. doi: 10.1105/tpc.20.00155 32409318 PMC7346570

[B53] Jores T. Tonnies J. Wrightsman T. Buckler E. S. Cuperus J. T. Fields S.. (2021). Synthetic promoter designs enabled by a comprehensive analysis of plant core promoters. Nat. Plants 7, 842–855. doi: 10.1038/s41477-021-00932-y 34083762 PMC10246763

[B54] Joshi R. Wani S. H. Singh B. Bohra A. Dar Z. A. Lone A. A.. (2016). Transcription factors and plants response to drought stress: current understanding and future directions. Front. Plant Sci. 7. doi: 10.3389/fpls.2016.01029 PMC4943945 27471513

[B55] Kabadi A. M. Gersbach C. A. (2014). Engineering synthetic TALE and CRISPR/Cas9 transcription factors for regulating gene expression. Methods 69, 188–197. doi: 10.1016/j.ymeth.2014.06.014 25010559 PMC4175060

[B56] Kar S. Bordiya Y. Rodriguez N. Kim J. Gardner E. C. Gollihar J. D.. (2022). Orthogonal control of gene expression in plants using synthetic promoters and CRISPR-based transcription factors. Plant Methods 18, 42. doi: 10.1186/s13007-022-00867-1 35351174 PMC8966344

[B57] Khadanga B. Chanwala J. Sandeep I. S. Dey N. (2021). Synthetic promoters from strawberry vein banding virus (SVBV) and Dahlia Mosaic Virus (DaMV). Mol. Biotechnol. 63, 792–806. doi: 10.1007/s12033-021-00344-5 34037929

[B58] Khakhar A. Leydon A. R. Lemmex A. C. Klavins E. Nemhauser J. L. (2018). Synthetic hormone-responsive transcription factors can monitor and re-program plant development. Elife 7, e34702. doi: 10.7554/eLife.34702 29714687 PMC5976440

[B59] Khan Z. H. Dang S. Memaya M. B. Bhadouriya S. L. Agarwal S. Mehrotra S.. (2022). Genome-wide analysis of AAAG and ACGT cis-elements in *Arabidopsis thaliana* reveals their involvement with genes downregulated under jasmonic acid response in an orientation independent manner. G3 12, jkac057. doi: 10.1093/g3journal/jkac057 35302624 PMC9073683

[B60] Khan A. Fornes O. Stigliani A. Gheorghe M. Castro-Mondragon J. A. van der Lee R.. (2018). JASPAR 2018: update of the open-access database of transcription factor binding profiles and its web framework. Nucleic Acids Res. 46, D260–D266. doi: 10.1093/nar/gkx1126 29140473 PMC5753243

[B61] Khan M. A. Herring G. Zhu J. Y. Oliva M. Fourie E. Johnston B.. (2024). CRISPRi-based circuits to control gene expression in plants. Nat. Biotechnol. 43, 416–430. doi: 10.1038/s41587-024-02236-w 38769424

[B62] Khan A. Nasim N. Pudhuvai B. Koul B. Upadhyay S. K. Sethi L.. (2023). Plant synthetic promoters: advancement and prospective. Agriculture 13, 298. doi: 10.3390/agriculture13020298

[B63] Kulakovskiy I. V. Medvedeva Y. A. Schaefer U. Kasianov A. S. Vorontsov I. E. Bajic V. B.. (2013). HOCOMOCO: a comprehensive collection of human transcription factor binding sites models. Nucleic Acids Res. 41, D195–D202. doi: 10.1093/nar/gks1089 23175603 PMC3531053

[B64] Lai X. Stigliani A. Vachon G. Carles C. Smaczniak C. Zubieta C.. (2019). Building transcription factor binding site models to understand gene regulation in plants. Mol. Plant 12, 743–763. doi: 10.1016/j.molp.2018.10.010 30447332

[B65] Lambrughi M. (2015). Network analysis and molecular dynamics simulations to investigate the link between structure and function in intrinsically disordered proteins and transcription factors.

[B66] Laxa M. Müller K. Lange N. Doering L. Pruscha J. T. Peterhänsel C. (2016). The 5′ UTR intron of Arabidopsis GGT1 aminotransferase enhances promoter activity by recruiting RNA polymerase II. Plant Physiol. 172, 313–327. doi: 10.1104/pp.16.00881 27418588 PMC5074633

[B67] Lee D. Hua L. Khoshravesh R. Giuliani R. Kumar I. Cousins A.. (2021). Engineering chloroplast development in rice through cell-specific control of endogenous genetic circuits. Plant Biotechnol. J. 19, 2291–2303. doi: 10.1111/pbi.13660 34328250 PMC8541780

[B68] Lescot M. Déhais P. Thijs G. Marchal K. Moreau Y. Van de Peer Y.. (2002). PlantCARE, a database of plant cis-acting regulatory elements and a portal to tools for in silico analysis of promoter sequences. Nucleic Acids Res. 30, 325–327. doi: 10.1093/nar/30.1.325 11752327 PMC99092

[B69] Levitsky V. Zemlyanskaya E. Oshchepkov D. Podkolodnaya O. Ignatieva E. Grosse I.. (2019). A single ChIP-seq dataset is sufficient for comprehensive analysis of motifs co-occurrence with MCOT package. Nucleic Acids Res. 47, e139–e139. doi: 10.1093/nar/gkz800 31750523 PMC6868382

[B70] Li J. Blue R. Zeitler B. Strange T. L. Pearl J. R. Huizinga D. H.. (2013). Activation domains for controlling plant gene expression using designed transcription factors. Plant Biotechnol. J. 11, 671–680. doi: 10.1111/pbi.12057 23521778

[B71] Li T. Huang S. Zhou J. Yang B. (2013). Designer TAL effectors induce disease susceptibility and resistance to Xanthomonas oryzae pv. oryzae in rice. Mol. Plant 6, 781–789. doi: 10.1093/mp/sst034 23430045

[B72] Li E. Liu H. Huang L. Zhang X. Dong X. Song W.. (2019). Long-range interactions between proximal and distal regulatory regions in maize. Nat. Commun. 10, 2633. doi: 10.1038/s41467-019-10603-4 31201330 PMC6572780

[B73] Li Z. Zhang D. Xiong X. Yan B. Xie W. Sheen J.. (2017). A potent Cas9-derived gene activator for plant and mammalian cells. Nat. Plants 3, 930–936. doi: 10.1038/s41477-017-0046-0 29158545 PMC5894343

[B74] Lieberman-Lazarovich M. Yahav C. Israeli A. Efroni I. (2019). Deep conservation of cis-element variants regulating plant hormonal responses. Plant Cell 31, 2559–2572. doi: 10.1105/tpc.19.00129 31467248 PMC6881130

[B75] Liu L. Gallagher J. Arevalo E. D. Chen R. Skopelitis T. Wu Q.. (2021). Enhancing grain-yield-related traits by CRISPR-Cas9 promoter editing of maize CLE genes. Nat. Plants 7, 287–294. doi: 10.1038/s41477-021-00858-5 33619356

[B76] Liu D. Hoynes-O’Connor A. Zhang F. (2013). Bridging the gap between systems biology and synthetic biology. Front. Microbiol. 4. doi: 10.3389/fmicb.2013.00211 PMC3722476 23898328

[B77] Lloyd J. P. Ly F. Gong P. Pflueger J. Swain T. Pflueger C.. (2022). Synthetic memory circuits for stable cell reprogramming in plants. Nat. Biotechnol. 40, 1862–1872. doi: 10.1038/s41587-022-01383- 35788565

[B78] Lowder L. G. Zhang D. Baltes N. J. Paul J. W. 3rd Tang X. Zheng X.. (2015). A CRISPR/cas9 toolbox for multiplexed plant genome editing and transcriptional regulation. Plant Physiol. 169, 971–985. doi: 10.1104/pp.15.00636 26297141 PMC4587453

[B79] Lowder L. G. Zhou J. Zhang Y. Malzahn A. Zhong Z. Hsieh T. F.. (2018). Robust transcriptional activation in plants using multiplexed CRISPR-Act2. 0 and mTALE-Act systems. Mol. Plant 11, 245–256. doi: 10.1016/j.molp.2017.11.010 29197638

[B80] Lu Z. Marand A. P. Ricci W. A. Ethridge C. L. Zhang X. Schmitz R. J. (2019). The prevalence, evolution and chromatin signatures of plant regulatory elements. Nat. Plants 5, 1250–1259. doi: 10.1038/s41477-019-0548-z 31740772

[B81] Lupo A. Cesaro E. Montano G. Zurlo D. Izzo P. Costanzo P. (2013). KRAB-zinc finger proteins: a repressor family displaying multiple biological functions. Curr. Genom. 14, 268–278. doi: 10.2174/13892029113149990002 PMC3731817 24294107

[B82] Mahfouz M. M. Li L. Piatek M. Fang X. Mansour H. Bangarusamy D. K.. (2012). Targeted transcriptional repression using a chimeric TALE-SRDX repressor protein. Plant Mol. Biol. 78, 311–321. doi: 10.1007/s11103-011-9866-x 22167390 PMC3259320

[B83] Marand A. P. Chen Z. Gallavotti A. Schmitz R. J. (2021). A cis-regulatory atlas in maize at single-cell resolution. Cell 184, 3041–3055. doi: 10.1016/j.cell.2021.04.014 33964211

[B84] Martínez de Alba A. E. Moreno A. B. Gabriel M. Mallory A. C. Christ A. Bounon R.. (2015). In plants, decapping prevents RDR6-dependent production of small interfering RNAs from endogenous mRNAs. Nucleic Acids Res. 43, 2902–2913. doi: 10.1093/nar/gkv119 25694514 PMC4357720

[B85] Matys V. Fricke E. Geffers R. Gößling E. Haubrock M. Hehl R.. (2003). TRANSFAC^®^: transcriptional regulation, from patterns to profiles. Nucleic Acids Res. 31, 374–378. doi: 10.1093/nar/gkg108 12520026 PMC165555

[B86] Mehrotra R. Gupta G. Sethi R. Bhalothia P. Kumar N. Mehrotra S. (2011). Designer promoter: an artwork of cis engineering. Plant Mol. Boil 75, 527–536. doi: 10.1007/s11103-011-9755-3 21327513

[B87] Mehrotra R. Mehrotra S. (2010). Promoter activation by ACGT in response to salicylic and abscisic acids is differentially regulated by the spacing between two copies of the motif. J. Plant Physiol. 167, 1214–1218. doi: 10.1016/j.jplph.2010.04.005 20554077

[B88] Mehrotra R. Renganaath K. Kanodia H. Loake G. J. Mehrotra S. (2017). Towards combinatorial transcriptional engineering. Biotechnol. Adv. 35, 390–405. doi: 10.1016/j.bioteChadv.2017.03.006 28300614

[B89] Mehrotra R. Sethi S. Zutshi I. Bhalothia P. Mehrotra S. (2013). Patterns and evolution of ACGT repeat cis-element landscape across four plant genomes. BMC Genom. 14, 1–11. doi: 10.1186/1471-2164-14-203 PMC3622567 23530833

[B90] Mejía-Guerra M. K. Buckler E. S. (2019). A k-mer grammar analysis to uncover maize regulatory architecture. BMC Plant Biol. 19, 1–17. doi: 10.1186/s12870-019-1693-2 30876396 PMC6419808

[B91] Misra S. Ganesan M. (2021). The impact of inducible promoters in transgenic plant production and crop improvement. Plant Gene 27, 100300. doi: 10.1016/j.plgene.2021.100300

[B92] Morbitzer R. Römer P. Boch J. Lahaye T. (2010). Regulation of selected genome loci using *de novo*-engineered transcription activator-like effector (TALE)-type transcription factors. PNAS 107, 21617–21622. doi: 10.1073/pnas.1013133107 21106758 PMC3003021

[B93] Morita S. Horii T. Kimura M. Hatada I. (2020). Synergistic upregulation of target genes by TET1 and VP64 in the dCas9–suntag platform. Int. J. Mol. Sci. 21, 1574. doi: 10.3390/ijms21051574 32106616 PMC7084704

[B94] Narusaka Y. Nakashima K. Shinwari Z. K. Sakuma Y. Furihata T. Abe H.. (2003). Interaction between two cis-acting elements, ABRE and DRE, in ABA-dependent expression of *Arabidopsis* rd29A gene in response to dehydration and high-salinity stresses. Plant J. 34, 137–148. doi: 10.1046/j.1365-313X.2003.01708.x 12694590

[B95] Naseri G. Balazadeh S. Machens F. Kamranfar I. Messerschmidt K. Mueller-Roeber B. (2017). Plant-derived transcription factors for orthologous regulation of gene expression in the yeast Saccharomyces cerevisiae. ACS Synth. Biol. 6, 1742–1756. doi: 10.1021/acssynbio.7b00094 28531348

[B96] Ochoa-Fernandez R. Abel N. B. Wieland F. G. Schlegel J. Koch L. A. Miller J. B.. (2020). Optogenetic control of gene expression in plants in the presence of ambient white light. Nat. Methods 17, 717–725. doi: 10.1038/s41592-020-0868-y 32601426

[B97] Oliva R. Ji C. Atienza-Grande G. Huguet-Tapia J. C. Perez-Quintero A. Li T.. (2019). Broad-spectrum resistance to bacterial blight in rice using genome editing. Nat. Biotechnol. 37, 1344–1350. doi: 10.1038/s41587-019-0267-z 31659337 PMC6831514

[B98] Pachkov M. Balwierz P. J. Arnold P. Ozonov E. Van Nimwegen E. (2012). SwissRegulon, a database of genome-wide annotations of regulatory sites: recent updates. Nucleic Acids Res. 41, D214–D220. doi: 10.1093/nar/gks1145 23180783 PMC3531101

[B99] Pan C. Wu X. Markel K. Malzahn A. A. Kundagrami N. Sretenovic S.. (2021). CRISPR–Act3. 0 for highly efficient multiplexed gene activation in plants. Nat. Plants 7, 942–953. doi: 10.1038/s41477-021-00953-7 34168320

[B100] Pandey S. P. Singh A. P. Srivastava S. Chandrashekar K. Sane A. P. (2019). A strong early acting wound-inducible promoter, RbPCD1pro, activates cryIAc expression within minutes of wounding to impart efficient protection against insects. Plant Biotechnol. J. 17, 1458–1470. doi: 10.1111/pbi.13071 30623549 PMC6576099

[B101] Panigrahi A. O’Malley B. W. (2021). Mechanisms of enhancer action: the known and the unknown. Genome Biol. 22, 1–30. doi: 10.1186/s13059-021-02322-1 33858480 PMC8051032

[B102] Papikian A. Liu W. Gallego-Bartolomé J. Jacobsen S. E. (2019). Site-specific manipulation of Arabidopsis loci using CRISPR-Cas9 SunTag systems. Nat. Commun. 10, 729. doi: 10.1038/s41467-019-08736-7 30760722 PMC6374409

[B103] Park J. Dempewolf E. Zhang W. Wang Z.-Y. (2017). RNA-guided transcriptional activation via CRISPR/dCas9 mimics overexpression phenotypes in *Arabidopsis* . PloS One 12, e0179410. doi: 10.1371/journal.pone.0179410 28622347 PMC5473554

[B104] Peng A. Chen S. Lei T. Xu L. He Y. Wu L.. (2017). Engineering canker-resistant plants through CRISPR/Cas9-targeted editing of the susceptibility gene Cs LOB 1 promoter in citrus. Plant Biotechnol. J. 15, 1509–1519. doi: 10.1111/pbi.12733 28371200 PMC5698050

[B105] Peramuna A. Bae H. Rasmussen E. K. Dueholm B. Waibel T. Critchley J. H.. (2018). Evaluation of synthetic promoters in Physcomitrella patens. Biochem. Biophys. Res. Commun. 500, 418–422. doi: 10.1016/j.bbrc.2018.04.092 29660341

[B106] Piatek A. Ali Z. Baazim H. Li L. Abulfaraj A. Al-Shareef S.. (2015). RNA-guided transcriptional regulation in planta via synthetic dCas9-based transcription factors. Plant Biotechnol. J. 13, 578–589. doi: 10.1111/pbi.12284 25400128

[B107] Pokhilko A. Mas P. Millar A. J. (2013). Modelling the widespread effects of TOC1 signaling on the plant circadian clock and its outputs. BMC Syst. Biol. 7, 1–12. doi: 10.1186/1752-0509-7-23 23506153 PMC3614443

[B108] Rajeevkumar S. Anunanthini P. Sathishkumar R. (2015). Epigenetic silencing in transgenic plants. Front. Plant Sci. 10. doi: 10.3389/fpls.2015.00693 PMC4564723 26442010

[B109] Ranjan R. Patro S. Kumari S. Kumar D. Dey N. Maiti I. B. (2011). Efficient chimeric promoters derived from full-length and sub-genomic transcript promoters of Figwort mosaic virus (FMV). J. Biotechnol. 152, 58–62. doi: 10.1016/j.jbiotec.2011.01.015 21262279

[B110] Ranjan R. Patro S. Pradhan B. Kumar A. Maiti I. B. Dey N. (2012). Development and functional analysis of novel genetic promoters using DNA shuffling, hybridization and a combination thereof. PloS One 7, e31931. doi: 10.1371/journal.pone.0031931 22431969 PMC3303778

[B111] Rantasalo A. Kuivanen J. Penttilä M. Jäntti J. Mojzita D. (2018). Synthetic toolkit for complex genetic circuit engineering in Saccharomyces cerevisiae. ACS synthetic Biol. 7, 1573–1587. doi: 10.1021/acssynbio.8b00076 PMC6150731 29750501

[B112] Ren C. Li H. Liu Y. Li S. Liang Z. (2022). Highly efficient activation of endogenous gene in grape using CRISPR/dCas9-based transcriptional activators. Hortic. Res. 9, uhab037. doi: 10.1093/hr/uhab037 35039855 PMC8807946

[B113] Ricci W. A. Lu Z. Ji L. Marand A. P. Ethridge C. L. Murphy N. G.. (2019). Widespread long-range cis-regulatory elements in the maize genome. Nat. Plants 5, 1237–1249. doi: 10.1038/s41477-019-0547-0 31740773 PMC6904520

[B114] Rodríguez-Leal D. Lemmon Z. H. Man J. Bartlett M. E. Lippman Z. B. (2017). Engineering quantitative trait variation for crop improvement by genome editing. Cell 171, 470–480. doi: 10.1016/j.cell.2017.08.030 28919077

[B115] Roell M. S. Zurbriggen M. D. (2020). The impact of synthetic biology for future agriculture and nutrition. COBIOT 61, 102–109. doi: 10.1016/j.copbio.2019.10.004 31812911

[B116] Samadder P. Sivamani E. Lu J. Li X. Qu R. (2008). Transcriptional and post-transcriptional enhancement of gene expression by the 5′ UTR intron of rice rubi3 gene in transgenic rice cells. MGG 279, 429–439. doi: 10.1007/s00438-008-0323-8 18236078

[B117] Sánchez J. P. Ullman C. Moore M. Choo Y. Chua N. H. (2006). Regulation of Arabidopsis thaliana 4-coumarate:coenzyme-A ligase-1 expression by artificial zinc finger chimeras. Plant Biotechnol. J. 4, 103–114. doi: 10.1111/j.1467-7652.2005.00161.x 17177789

[B118] Schmitz R. J. Grotewold E. Stam M. (2022). Cis-regulatory sequences in plants: Their importance, discovery, and future challenges. Plant Cell 34, 718–741. doi: 10.1093/plcell/koab281 34918159 PMC8824567

[B119] Shahmuradov I. A. Umarov R. K. Solovyev V. V. (2017). TSSPlant: a new tool for prediction of plant Pol II promoters. Nucleic Acids Res. 45, e65–e65. doi: 10.1093/nar/gkw1353 28082394 PMC5416875

[B120] Shen Q. Zhang P. Ho T. H. (1996). Modular nature of abscisic acid (ABA) response complexes: composite promoter units that are necessary and sufficient for ABA induction of gene expression in barley. Plant Cell 8, 1107–1119. doi: 10.1105/tpc.8.7.1107 8768371 PMC161185

[B121] Shikata H. Denninger P. (2022). Plant optogenetics: applications and perspectives. Curr. Opin. Plant Biol. 68, 102256. doi: 10.1016/j.pbi.2022.102256 35780691

[B122] Shrestha A. Khan A. Dey N. (2018). Cis–trans engineering: advances and perspectives on customized transcriptional regulation in plants. Mol. Plant 11, 886–898. doi: 10.1016/j.molp.2018.05.008 29859265

[B123] Sidorenko L. V. Lee T. F. Woosley A. Moskal W. A. Bevan S. A. Merlo P. A. O.. (2017). GC-rich coding sequences reduce transposon-like, small RNA-mediated transgene silencing. Nat. Plants 3, 875–884. doi: 10.1038/s41477-017-0040-6 29085072

[B124] Sinha S. Adler A. S. Field Y. Chang H. Y. Segal E. (2008). Systematic functional characterization of cis-regulatory motifs in human core promoters. Genome Res. 18, 477–488. doi: 10.1101/gr.6828808 18256240 PMC2259112

[B125] Slattery M. Zhou T. Yang L. MaChado A. C. D. Gordân R. Rohs R. (2014). Absence of a simple code: how transcription factors read the genome. Trends Biochem. Sci. 39, 381–399. doi: 10.1016/j.tibs.2014.07.002 25129887 PMC4149858

[B126] Sleight S. C. Bartley B. A. Lieviant J. A. Sauro H. M. (2010). Designing and engineering evolutionary robust genetic circuits. J. Biol. Eng. 4, 1–20. doi: 10.1186/1754-1611-4-12 21040586 PMC2991278

[B127] Sun J. He N. Niu L. Huang Y. Shen W. Zhang Y.. (2019). Global quantitative mapping of enhancers in rice by STARR-seq. Genom. Proteom. Bioinf. 17, 140–153. doi: 10.1016/j.gpb.2018.11.003 PMC6624190 31201999

[B128] Szenk M. Yim T. Balázsi G. (2020). Multiplexed gene expression tuning with orthogonal synthetic gene circuits. ACS Synth. Biol. 9, 930–939. doi: 10.1021/acssynbio.9b00534 32167761 PMC7197936

[B129] Tang H. Wu Y. Deng J. Chen N. Zheng Z. Wei Y.. (2020). Promoter architecture and promoter engineering in Saccharomyces cerevisiae. Metabolites 10, 320. doi: 10.3390/metabo10080320 32781665 PMC7466126

[B130] Tian F. Yang D. C. Meng Y. Q. Jin J. Gao G. (2020). PlantRegMap: charting functional regulatory maps in plants. Nucleic Acids Res. 48, D1104–D1113. doi: 10.1093/nar/gkz1020 31701126 PMC7145545

[B131] Tol Nv. Andaluz G. F. Leeggangers H. A. C. F. Roushan M. R. Hooykaas P. J. J.. (2019). Zinc finger artificial transcription factor-mediated chloroplast genome interrogation in *arabidopsis thaliana* . Plant Cell Physiol. 60, 393–406. doi: 10.1093/pcp/pcy216 30398644 PMC6375250

[B132] Tycko J. DelRosso N. Hess G. T. Banerjee A. Mukund A. Van M. V.. (2020). High-throughput discovery and characterization of human transcriptional effectors. Cell 183, 2020–2035. doi: 10.1016/j.cell.2020.11.024 33326746 PMC8178797

[B133] Uniyal A. P. Mansotra K. Yadav S. K. Kumar V. (2019). An overview of designing and selection of sgRNAs for precise genome editing by the CRISPR-Cas9 system in plants. 3 Biotech. 9, 223. doi: 10.1007/s13205-019-1760-2 PMC6529479 31139538

[B134] Uygun S. Azodi C. B. Shiu S. H. (2019). Cis-regulatory code for predicting plant cell-type transcriptional response to high salinity. Plant Physiol. 181, 1739–1751. doi: 10.1104/pp.19.00653 31551359 PMC6878017

[B135] Vaishnav E. D. deBoer C. G. Molinet J. Yassour M. Fan L. Adiconis X.. (2022). The evolution, evolvability, and engineering of gene regulatory DNA. Nature 603, 455–463. doi: 10.1038/s41586-022-04506-6 35264797 PMC8934302

[B136] Vanaja A. Yella V. R. (2022). Delineation of the DNA structural features of eukaryotic core promoter classes. ACS Omega 7, 5657–5669. doi: 10.1021/acsomega.1c04603 35224327 PMC8867553

[B137] Van Bel M. Diels T. Vancaester E. Kreft L. Botzki A. Van de Peer Y.. (2018). PLAZA 4.0: an integrative resource for functional, evolutionary and comparative plant genomics. Nucleic Acids Res. 46, D1190–D1196. doi: 10.1093/nar/gkx1002 29069403 PMC5753339

[B138] Van Eenennaam A. L. Li G. Venkatramesh M. Levering C. Gong X. Jamieson A. C.. (2004). Elevation of seed alpha-tocopherol levels using plant-based transcription factors targeted to an endogenous locus. Metab. Eng. 6, 101–108. doi: 10.1016/j.ymben.2003.11.001 15113563

[B139] Wagner A. (2022). AI predicts the effectiveness and evolution of gene promoter sequences. Nature 603, 399–400. doi: 10.1038/d41586-022-00384-0 35264799

[B140] Wang H. Cimen E. Singh N. Buckler E. (2020). Deep learning for plant genomics and crop improvement. Curr. Opin. Plant Biol. 54, 34–41. doi: 10.1016/j.pbi.2019.12.010 31986354

[B141] Wang X. Hu Z. Yu T. Wang Y. Wang R. Wei Y.. (2023). Con-AAE: contrastive cycle adversarial autoencoders for single-cell multi-omics alignment and integration. Bioinformatics 39, btad162. doi: 10.1093/bioinformatics/btad162 36975610 PMC10101696

[B142] Weirauch M. T. Yang A. Albu M. Cote A. G. Montenegro-Montero A. Drewe P.. (2014). Determination and inference of eukaryotic transcription factor sequence specificity. Cell 158, 1431–1443. doi: 10.1016/j.cell.2014.08.009 25215497 PMC4163041

[B143] Whitington T. Frith M. C. Johnson J. Bailey T. L. (2011). Inferring transcription factor complexes from ChIP-seq data. Nucleic Acids Res. 39, e98–e98. doi: 10.1093/nar/gkr341 21602262 PMC3159476

[B144] Wu M. R. Nissim L. Stupp D. Pery E. Binder-Nissim A. Weisinger K.. (2019). A high-throughput screening and computation platform for identifying synthetic promoters with enhanced cell-state specificity (SPECS). Nat. Commun. 10, 2880. doi: 10.1038/s41467-019-10912-8 31253799 PMC6599391

[B145] Xiong X. Liang J. Li Z. Gong B. Q. Li J. F. (2021). Multiplex and optimization of dCas9-TV-mediated gene activation in plants. J. Integr. Plant Biol. 63, 634–645. doi: 10.1111/jipb.13023 33058471

[B146] Yamashita R. Sathira N. P. Kanai A. Tanimoto K. Arauchi T. Tanaka Y.. (2011). Genome-wide characterization of transcriptional start sites in humans by integrative transcriptome analysis. Genome Res. 21, 775–789. doi: 10.1101/gr.110254.110 21372179 PMC3083095

[B147] Yang Y. Al-Baidhani H. H. J. Harris J. Riboni M. Li Y. Mazonka I.. (2020). DREB/CBF expression in wheat and barley using the stress-inducible promoters of HD-Zip I genes: impact on plant development, stress tolerance and yield. Plant Biotechnol. J. 18, 829–844. doi: 10.1111/pbi.13252 31487424 PMC7004899

[B148] Yang Y. Lee J. H. Poindexter M. R. Shao Y. Liu W. Lenaghan S. C.. (2021). Rational design and testing of abiotic stress-inducible synthetic promoters from poplar cis-regulatory elements. Plant Biotechnol. J. 19, 1354–1369. doi: 10.1111/pbi.13550 33471413 PMC8313130

[B149] Yasmeen E. Wang J. Riaz M. Zhang L. Zuo K. (2023). Designing artificial synthetic promoters for accurate, smart, and versatile gene expression in plants. Plant Commun. 4, 100558 . doi: 10.1016/j.xplc.2023.100558 36760129 PMC10363483

[B150] Yun A. Kang J. Lee J. Song S. J. Hwang I. (2023). Design of an artificial transcriptional system for production of high levels of recombinant proteins in tobacco (*Nicotiana benthamiana*). Front. Plant Sci. 14, 1138089. doi: 10.3389/fpls.2023.1138089 36909433 PMC9995837

[B151] Zalatan J. G. Lee M. E. Almeida R. Gilbert L. A. Whitehead E. H. La Russa M.. (2015). Engineering complex synthetic transcriptional programs with CRISPR RNA scaffolds. Cell 160, 339–350. doi: 10.1016/j.cell.2014.11.052 25533786 PMC4297522

[B152] Zemlyanskaya E. V. Dolgikh V. A. Levitsky V. G. Mironova V. (2021). Transcriptional regulation in plants: Using omics data to crack the cis-regulatory code. Curr. Opin. Plant Biol. 63, 102058. doi: 10.1016/j.pbi.2021.102058 34098218

[B153] Zetsche B. Gootenberg J. S. Abudayyeh O. O. Slaymaker I. M. Makarova K. S. Essletzbichler P.. (2015). Cpf1 is a single RNA-guided endonuclease of a class 2 CRISPR-Cas system. Cell 163, 759–771. doi: 10.1016/j.cell.2015.09.038 26422227 PMC4638220

[B154] Zhang T. Q. Xu Z. G. Shang G. D. Wang J. W. (2019). A single-cell RNA sequencing profiles the developmental landscape of *Arabidopsis* root. Mol. Plant 12, 648–660. doi: 10.1016/j.molp.2019.04.004 31004836

[B155] Zhang X. Zhu Y. Liu X. Hong X. Xu Y. Zhu P.. (2015). Suppression of endogenous gene silencing by bidirectional cytoplasmic RNA decay in *Arabidopsis* . Science 348, 120–123. doi: 10.1126/science.aaa2618 25838384

